# Rapid Screening of 350 Pesticide Residues in Vegetable and Fruit Juices by Multi-Plug Filtration Cleanup Method Combined with Gas Chromatography-Electrostatic Field Orbitrap High Resolution Mass Spectrometry

**DOI:** 10.3390/foods10071651

**Published:** 2021-07-16

**Authors:** Zhijuan Meng, Qiang Li, Jianhan Cong, Yunxia Huang, Dong Wang, Canping Pan, Sufang Fan, Yan Zhang

**Affiliations:** 1 Key Laboratory of Food Safety of Hebei Province, Hebei Food Inspection and Research Institute, Shijiazhuang 050091, China; zjmengv1@163.com (Z.M.); liqiang@nepp.com.cn (Q.L.); huangyunxia@nepp.com.cn (Y.H.); wd3199@139.com (D.W.); 2 Beijing Key Laboratory of Environmental and Viral Oncology, Faculty of Environment and Life, Beijing University of Technology, Beijing 100124, China; 15732636263@163.com; 3 Department of Applied Chemistry, College of Science, China Agricultural University, Beijing 100193, China; canpingp@cau.edu.cn

**Keywords:** GC-Orbitrap/MS, m-PFC, pesticide residues, rapid screening, vegetable and fruit juices

## Abstract

A new method for screening pesticide residues in vegetable and fruit juices by the multi-plug filtration cleanup (m-PFC) method combined with gas chromatography-electrostatic field orbitrap high resolution mass spectrometry(GC-Orbitrap/MS) was developed. The samples were extracted with acetonitrile, purified with m-PFC and determined by GC-Orbitrap/MS. Qualitative analysis was confirmed by retention time, accurate molecular mass and quantitative analysis were performed with the matrix standard calibration. It could eliminate matrix interference effectively. Eight kinds of typical samples (orange juice, apple juice, grape juice, strawberry juice, celery juice, carrot juice, cucumber juice, tomato juice) were evaluated. The linear ranges of the 350 pesticides were from 5 to 500 μg/kg, with good correlation coefficients greater than 0.990. The limits of detection (LODs) were 0.3–3.0 μg/kg and the limits of quantification (LOQs) were 1.0–10.0 μg/kg. The average recoveries at three spiked levels of 10, 100, 200 μg/kg were in the range of 72.8–122.4%, with relative standard deviations (RSDs) of 2.0–10.8%. The method has effectively improved the determination efficiency of pesticide residue screening by high-resolution mass spectrometry in vegetable and fruit juices.

## 1. Introduction

With the deepening of the concept of health, consumers pay more attention to health.They give up carbonated drinks and turn to fruit and vegetable juice drinks. Fruit and vegetable juice drinks are favored for their rich nutrition, good health effects and safety. They account for about 24 percent of the global beverage market and have become a popular drink. However, the improper use of different kinds of pesticides, and illegal use of prohibited and restricted pesticides in the production of fruits and vegetables are becoming more and more serious. Excessive pesticide residues in fruits and vegetables often occur, which indirectly leads to a variety of pesticide residues in fruit and vegetable juice [1]. As a result, the ecological environment and human health are endangered. The phenomenon of multiple pesticide residues in fruit and vegetable juice is prominent. The problem of pesticide residues has become one of the major food safety issues of international concern. Many countries and international organizations (such as the United States, Japan, China, the European Union and South Korea, etc.) have established maximum residue limits (MRLs) for pesticide residues in fruit and vegetable juices. The limit level of some pesticide compounds is as low as 10 μg/kg, and shows a trend of becoming stricter year by year [2]. The implementation of these standards strengthens the supervision of pesticides, ensures the standardized use of pesticides and avoids trade disputes. With the increasing consumption and export of fruit and vegetable juice, the state has increased the monitoring of pesticide residues in fruit and vegetable juice. At present, scholars in various countries mainly focus on the analysis of pesticide residues in fruits and vegetables, but there are relatively few studies on pesticide residues in fruit juice and vegetable juice. Therefore, it is necessary to establish a rapid, effective and sensitive method for the analysis of pesticide residues in fruit and vegetable juice [3,4,5].

**Figure 1:**
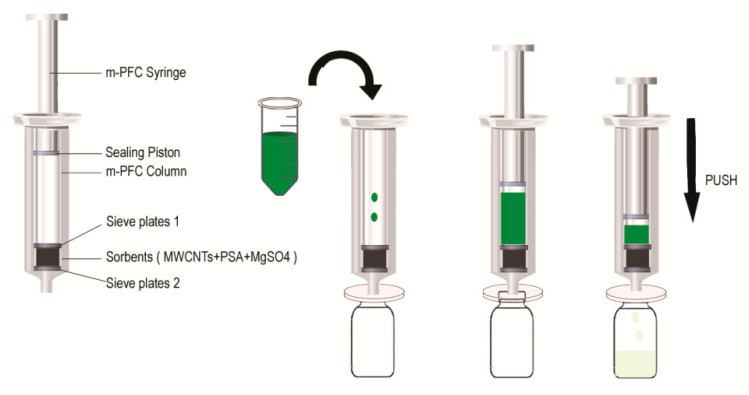
Schematic diagram of multi-plug filtration cleanup (m-PFC) syringe and cleanup procedure diagram.

Pesticide residue analysis is a complex trace analysis technique, in which the pretreatment method plays an important role in the detection process. The traditional extraction and purification technology could no longer meet the requirements of modern pesticide residue analysis, especially when the food matrix is becoming more and more complex [6,7,8]. Proper pretreatment technology can improve the sensitivity, detection range, precision and accuracy of detection [9,10,11]. At present, sample pretreatment methods include solid phase extraction [12,13], solid phase microextraction [14], gel permeation chromatography [15,16] and QuEChERS [17,18,19], etc. The QuEChERS method is a simple and rapid pretreatment technology for pesticide residues. Its principle is to use adsorbents to adsorb impurities to achieve the purpose of purification. It is the method certified by the American official Association of Analytical Chemists (AOAC2007.01) and the European Standard method certified by the European Committee for Standardization (EN15662-2018) [20,21]. m-PFC is a new rapid sample pretreatment method based on the QuEChERS method. As shown in Figure 1, this method places packing in the syringe. The extraction solution is pushed through the packing layer containing multi-walled carbon nanotubes (MWCNTs), primary secondary amine (PSA) and MgSO_4_. The filler reacts or adsorbs with the interfering substances in the matrix, such as pigments, lipids, some sugars, sterols, tea polyphenols, organic acids and alkaloids, but does not react with the target compound. By realizing one-step purification, the purification time is greatly shortened and the pretreatment efficiency can be greatly improved. It has been applied to the detection of multi-pesticide residues in ginseng, agricultural products, fruits and vegetables [22,23,24,25,26,27,28]. The main detection methods are gas chromatography-mass spectrometry (GC-MS/MS) and liquid chromatography-mass spectrometry (LC-MS/MS) [19,29,30,31,32,33].

When the sample matrix is complex, the target and interference with similar mass numbers cannot be distinguished effectively. The traditional triple quadrupole mass spectrometry often has false positives. At the same time, when using GC-MS/MS and LC-MS/MS usually have the problems of false positive, it is often necessary to divide the compounds into several groups for separate detection, which greatly limits the application of this method. Besides, qualitative analysis of GC-MS/MS and LC-MS/MS must rely on a reference, which increases the cost of the experiment and the workload of analysis. GC-Orbitrap/MS has the advantages of fast analysis speeds, high resolution and quality accuracy. The determination of compounds is not limited by quantity, and more comprehensive information of compounds can be collected. The resolution of more than 60.000 (*m*/*z* 200) is selected for the parameters, which greatly improves the speed of qualitative screening for pesticide residues and the reliability of the results. At the same time, more accurate quantitative results can be obtained, which can be used for screening and quantitative analysis of low-level target compounds in the complex matrix. The newly added compounds can be analyzed retroactively, which greatly increases the number of synchronous screenings of compounds. At present, it has been used in fruits and vegetables [34,35], grain and feed [36], and fruit and vegetable baby food [37].To our knowledge, the application of m-PFC purification combined with GC-Orbitrap/MS technology in the determination of multi-pesticide residues in fruit and vegetable juice has not been reported.

In our previous study, an m-PFC cleanup method combined with GC-TOF/MS for vegetables and fruits has been developed [28]. Though the method has been developed for vegetables and fruits samples analysis, validation of the method for vegetable and fruit juices has not been performed. Therefore, in the work presented, the m-PFC cleanup method for eight vegetables and fruits samples were evaluated, along with GC-Orbitrap/MS detection in order to monitor the quality of fruit and vegetable juices in China. The objective of this study was to develop and validate cleanup methods for the detection and quantification of multi-residues in vegetable and fruit juices. The actual sample verification shows that this method is suitable for rapid screening and analysis of pesticide residues in a large number of samples.

## 2. Materials and Methods

### 2.1. Chemicals and Reagents

Analytical reference standards of the 350 pesticides were purchased from Alta Scientific Co., Ltd. Stock solutions (Tianjin, China) and were stored at −20 °C. Internal standards of heptachlor epoxide were purchased from Aldrich-Sigma (Shanghai, China). 

HPLC grade acetonitrile (MeCN) was purchased from Merck (Darmstadt, Germany). 

Two types of m-PFC were provided by China Agricultural University (Beijing, China). m-PFC cartridge (simple matrix): 15 mg MWCNTs + 15 mg PSA + 150 g MgSO_4_; m-PFC (complex matrix): 25 mg MWCNTs + 15 mg PSA + 150 g MgSO_4_. Two types of traditional QuEChERS purification package were purchased from Thermo Fisher Scientific (Beverly, MA, USA). QuEChERS (simple matrix): 50.0 mg PSA + 150.0 mg MgSO_4_; QuEChERS (complex matrix): 50.0 mg PSA + 150.0 mg MgSO_4_ + 50 mg C_18_ + 50 mg GCB. Three kinds of salting out packages for the QuEChERS method: 4 g MgSO_4_, 1 g NaCl; AOAC 2007.01 Method: 6 g MgSO_4_, 1.5 g sodium acetate (NaOAc); EN15662 Method: 4 g MgSO_4_, 1 g NaCl, 1 g trisodium citrate dihydrate (TSCD), 0.5 g disodium hydrogen sesquihydrate (DHS) respectively were provided by Lumiere Technologies (Beijing, China).

### 2.2. Stock Solutions and Standards

Stock solutions were prepared in a suitable solvent (typically ethyl acetate or acetonitrile) at a concentration of 100 μg/mL and were stored in amber screw-capped glass vials in the darkness at −20 °C. From the stock solution mix-standards were prepared in acetonitrile at 10 μg/mL. Further dilutions were prepared in ethyl acetate and used for the calibration as needed. Injection internal standard (heptachlor epoxide) was added to all prepared vials at 50 μg/kg final concentration. Linearity was studied for the 350 analytes at five calibration levels (0.005, 0.02, 0.05, 0.2, 0.5 μg/mL) by matrix-matched standard calibration in each blank matrix extracts. Mixed multi-standard working solutions were freshly prepared to avoid the degradation of pesticides by serial dilution.

### 2.3. GC-Orbitrap Analytical Conditions

A GC-Orbitrap system (Thermo Scientific, Bremen, Germany) consisting of an AI/AS 1310 autosampler, a TRACE 1300 Series GC with a hot split/splitless injector, an electron impact ion source (EI), and a hybrid quadrupole Orbitrap mass spectrometer with an HCD (higher energy collision-induced dissociation) cell was used. The column was set at a constant flow rate of 1.0 mL/min using helium as carrier gas (purity ≥ 99.999%). GC separation was performed on a 30 m × 0.25 mm ID, 0.25 μm TG-5MS column (5% phenyl-95% methylpolysiloxane, Thermo Scientific). The column temperature program started from 40 °C (hold 1.5 min), increased to 90 °C at the rate of 25 °C/min, then increased to 180 °C at the rate of 25 °C/min, then increased to 280 °C at the rate of 5 °C/min, then increased to 310 °C at the rate of 10 °C/min, and held at this final temperature for 3 min. The temperature of the injector port was 270 °C, and a 1 µL volume was injected into the splitless mode. The helium carrier gas flow rate was 1.0 mL/min. EI was performed at 70 eV, with the ion source and transfer line temperature at 280 °C.

Full scan MS acquisition was done in profile mode using a *m*/*z* range of 50–500. The nitrogen gas supply for the C-trap and HCD cell was 5.0 grade (99.999%) (Linde Gas, Shanghai, China). For the evaluation of the system, different settings for resolving power [15,000; 30,000; 60,000; and 120,000 full width at half maxima (FWHM) at *m*/*z* 200] and automatic gain control (AGC) target values (2 × 10^5^, 1 × 10^6^, and 3 × 10^6^) were tested. In the final method, the Orbitrap resolving power was set at 60,000 and the AGC target at 3E6. The actual scan speed under these conditions was approximately 4 scans/s.External mass calibration was performed before each sequence using perfluorotributylamine (68.9945, 99.9928, 130.9911, 196.9827, 218.9846, 263.9860, 413.9760, and 501.9694) with a mass error tolerance of ±1 ppm (±0.2 mDa), and during the measurement internal mass calibration was carried out by the instrument using three background ions from the column bleed as lock mass (C_5_H_15_O_3_Si_3_^+^, 207.03236; C_7_H_21_O_4_Si_4_^+^, 281.05115; C_9_H_27_O_5_Si_5_^+^, 355.06994) with a search window of ±2 ppm (±1 mDa). If in a certain scan none of the three specified background ions were found within ±2 ppm of their exact mass, no internal locking was applied for that scan. The instrument was controlled using Tune 2.8 and TraceFinder 4.1 (Thermo Scientific).

### 2.4. Sample Preparation

Vegetable and fruit juice samples were collected from supermarkets and farmers’ markets in different cities of Hebei Province. Samples were homogenized and filtered before extraction to remove the sediments. The prepared samples were stored at 4 °C and analyzed within 24 h following the procedure described below. Vegetable and fruit juice samples (pesticide-free) obtained from an organic production base was used as blank matrixes for preparing the standard curve and the recovery studies.

Extraction method: Method (1) In this study, an amount (10.00 ± 0.01 g) of vegetable and fruit juice samples were weighed into a 50 mL centrifuge tube, and 10 mL of acetonitrile was added. The resulting solution was shaken by the vortex for 1 min. After following traditional QuEChERS extraction a salt pack was added, the tube was cooled in an ice-water bath immediately. The centrifuge tube was shaken vigorously for an additional 1 min. The extract was then centrifuged at 9500 rpm/min for 3 min. Method (2) The amount (15.00 ± 0.01 g) of vegetable and fruit juice samples were weighed and 15 mL of acetonitrile was added. AOAC 2007. 01 extraction salt pack (6 g MgSO_4_, 1.5 g NaOAc) was added, other operations were the same as the Method (1). Method (3), EN15662 extraction salt pack (4 g MgSO_4_, 1 g NaCl, 1 g TSCD) was used, other operations were the same as the Method (1).

Purification method: Method (1) After centrifugation, 2 mL upper acetonitrile layer was transferred to the QuEChERS purification centrifuge tube. The mixture was vortexed for 1 min and centrifuged at 9500 rpm/min for 3 min. Before GC-Orbitrap analysis, the solution was filtered through a 0.22 µm filter (Tianjin, China). Method (2) The supernatant was used for further m-PFC procedures; 2 mL of supernatant was added from the top of the m-PFC purification column, and the plunger rod was slowly pushed (1–1.5 s/drop). Before GC-Orbitrap analysis, the solution was filtered through a 0.22 µm filter.

### 2.5. Database for Screening, Qualitative and Quantitative Rules

In this experiment, 350 pesticide compounds were selected and prepared into a mixed standard solution of 1.0 μg/mL. For data processing in TraceFinder 4.1, exact masses of three ions (quantifier and qualifier) for each compound were selected. The list of analytes and their retention times, chemical abstracts service (CAS) numbers, formulas and the exacts masses of the ions werepresented in Supplementary Materials Table S1. The database was organized and built in Tracefinder, whichcould not only realize fast, batch and automatic data processing, but also set the functions of qualitative, quantitative and method establishment. According to the established database, it can realize the rapid screening of target compounds.

## 3. Results

### 3.1. Optimization and Comparison of the Extraction Procedure

In this paper, 350 kinds of pesticides were selected as target analysis compounds according to the catalog of pesticides involved in the national random inspection and the routine inspection project of the Ministry of Agriculture of China. It also combined with the types of pesticides commonly used in fruit and vegetable planting in China. When methanol was used as the extraction solvent, less pigment was extracted, but the recovery was in range of 0–56%. Using ethyl acetate extraction, the recovery rates of some organophosphorus pesticides, such as heptachloride, were less than 70%.The pesticides contain organophosphorus, organochlorine, pyrethroid, triazole, etc., which contain many types and polarities. So, it is particularly important to choose a suitable extraction solvent. Acetonitrile has good solubility, strong penetration, versatility, high extraction efficiency, and effective reduction of interference from oils. Considering all the above reasons, acetonitrile was chosen as extraction solution. In this study, three versions of buffer salts were compared, and the extraction efficiency was evaluated using three levels (10, 100, 200 µg/kg) spiked recovery in orange juice samples. The results showed that most pesticides, except for pH-sensitive ones, gave excellent results when extracted with three different versions of buffer salts. The QuEChERS version using acetate buffering or citrate buffering more often gave higher recoveries compared with the unbuffered method for pH-dependent pesticides. Acephate, dimethoate, methamidophos, omethoate and profenofos are unstable in alkaline medium. It is easier to achieve higher recovery in a buffer salt system with the pH of the matrix being maintained between 5.0 and 6.0 throughout the experiment. This is suitable for a variety of fruit and vegetable juice samples. At the same time, the recovery rateof pesticides in acetate buffer version (AOAC) is slightly higher than that in citrate buffer version (EN), but the difference is not obvious. Therefore, acetonitrile was selected as the extraction solvent and acetic acid buffer salt was added to adjust the pH valuein this experiment. The acetic acid buffer salt system contains anhydrous MgSO_4_, which is exothermic when absorbed water in the extraction process. Some organophosphoruspesticides, such as methamidophos, parathion and fenitrothion are chemically unstable and easily decompose at high temperature with a recovery rate of less than 70%. In the ice-water bath, the recovery of these compounds was more than 70%, which met the requirements. Therefore, after adding the acetic acid buffer salt, the tube was cooled in an ice-water bath immediately for 2 min to reduce the recovery rate of pesticides with poor thermal stability.

### 3.2. Optimization and Comparison of the Cleanup Procedure

**Figure 2:**
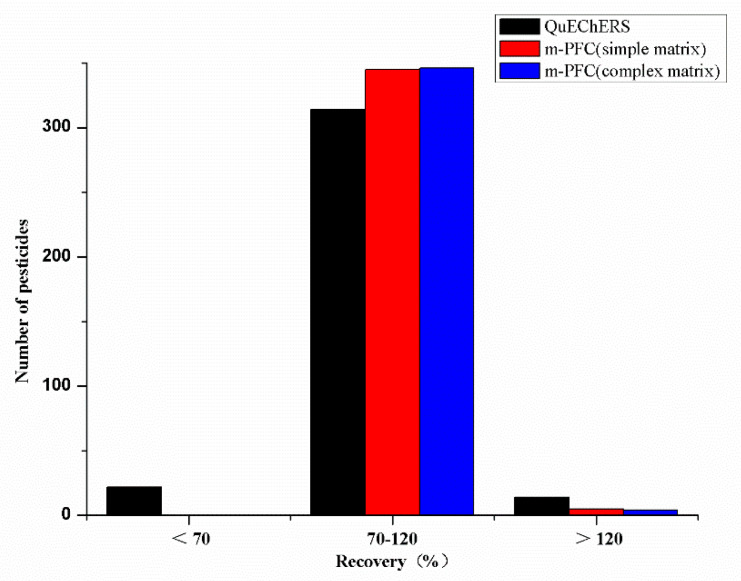
Recoveries of 350 pesticides in the orange juice samples cleaned by differentpurification methods (100 μg/kg).

Fruit and vegetable juice matrixes contain lipids, pigments, sugars and organic acids, which will be extracted together with the target substance in the extraction process. Without further purification, the target will be disturbed and the qualitative and quantitative results will be affected. Traditional QuEChERS cleanup and m-PFC cleanup were compared. The traditional QuEChERS mainly works with PSA. For pigmented fruits and vegetables GCB is added. PSA provided polaradsorption and weak anion exchange, which removed acidinterfering substances and some polar pigments such as fattyacids, phenols, and carbohydrates. However, the clean-up performance of PSA was not enough to remove interfering substances in studied matrices. GCBpossesseda special chemical structure, which adsorbed non-polar interferences such as pigments and sterols effectively. However, it has a strong adsorption effect on compounds containing benzene ring functional groups. The m-PFC method basing on QuEChERS was developed. The m-PFC column simplifies the pretreatment operation and takes advantage of MWCNTs (particle size length: 10–50 μm, outer diameter: 30–60 nm, specific surface area: 280 m^2^/g). MWCNTs are a new composite nanomaterial which can be used to remove pigments from the matrices. Owing to their extremely large surface area and unique structure, MWCNTs have excellent adsorption abilities compared to other sorbents. MWCNTs can remove pigments, organic acids, some sugars, esters, sterols and other interfering substances in the matrix effectively, and improve the adsorption problem of GCB in some planar structure compounds. The recovery rates of 350 pesticides in orange juice samples were determined by using m-PFC column and traditional QuEChERS purification at spiked levels of 100 μg/kg. By comparison, the number of pesticides withrecoveries between 70% to 120% were greater when using the m-PFC method, as shown in Figure 2. The recoveries of all pesticides were 73.2–122.4% with RSDs lower than 10.8% by m-PFC cleanup. Spike recoveries were within 63.2 and 123.5% and the RSDs were less than 15% using the traditional QuEChERS cleanup. The recovery rates of the two methods are different, mainly due to the adsorption in the GCB. So, m-PFC cleanup is more efficient than the traditional QuEChERS cleanup. Meanwhile, it has no effect on the target recovery rate. For complex substrates with severe pigmentation (such as celery juice and cucumber juice), m-PFC (complex substrates) was used. Compared with m-PFC small column (simple matrix), the purification effect was better when 15 mg MWCNTs were adjusted to 25 mg MWCNTs, and other materials were not changed. The recoveries of all pesticides ranged from 72.8% to 121.3% with RSDs less than 10.4%, which could meet the screening requirements. There was no significant difference compared with m-PFC (simple matrix), but it could reduce the pollution and damage the analytical instrument. Compared with the traditional QuEChERS method, the purification process was less than 2 min, and has no vortices or centrifugation. The purification capacity was enhanced, the matrix effect was eliminated, and the maintenance period of the analytical instrument was prolonged. Therefore, m-PFC column was selected for purification in this experiment.

### 3.3. Optimization of Instrument Resolution

**Figure 3:**
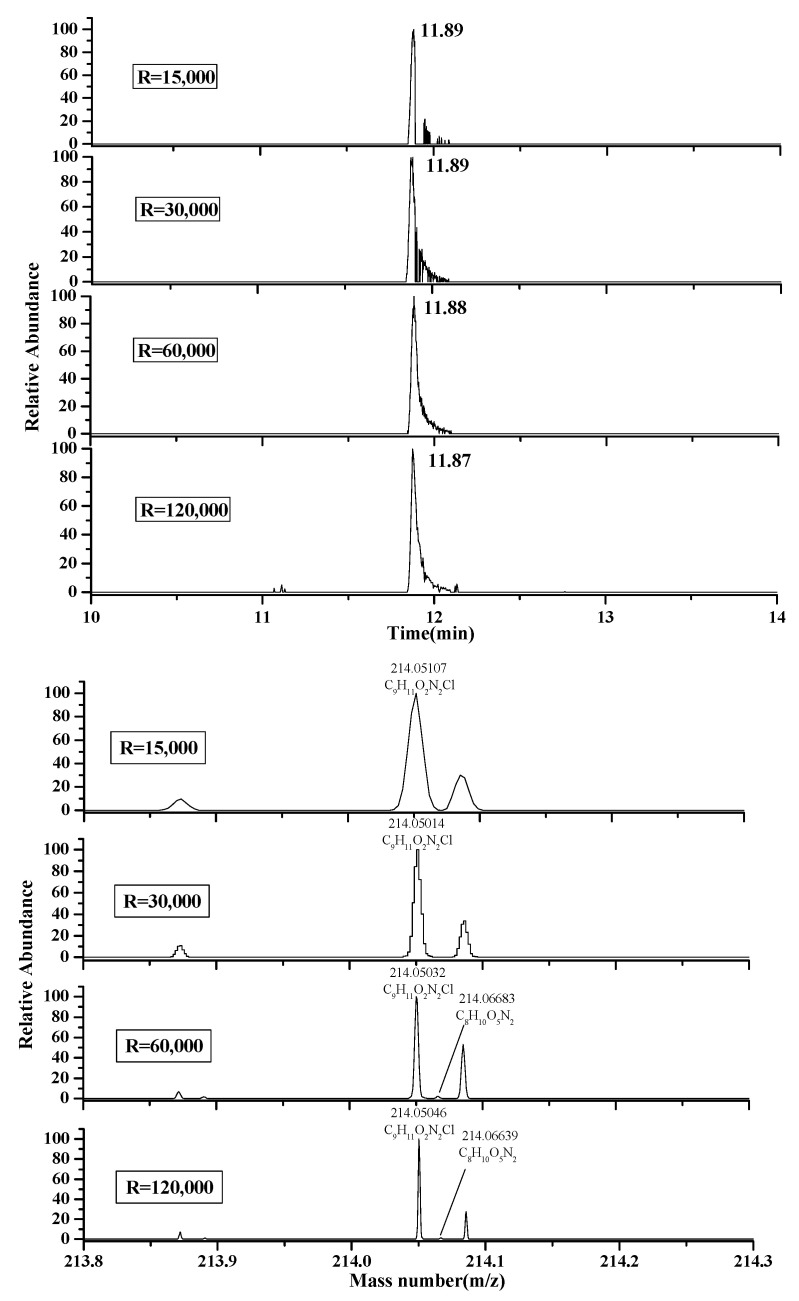
Extracted ion chromatograms andquality accuracy of the spiked (10 μg/kg) monolinuron qualitative ion (214.0504) in the orange juice sample at different resolutions.

Resolution is a key parameter in high-resolution mass spectrometry (HRMS). It minimizes interference with coeluting matrixes and other target compounds, avoiding inadequate mass errors due to the of merging isobaric ions in the analysis of fruit and vegetable juice samples. The resolution values selected and evaluated were 15,000, 30,000, 60,000 and 120,000 FWHM at *m*/*z* 200. In theory, the high resolution is a better option considering the coeluting isobariccompounds, but a higher resolution will lead to lower scan speeds and therefore reduces the number of points across a peak. At 120,000 the scan speed is around 3.5 scans per second. A good compromise to obtain optimum results is the selection of 60,000 FWHM of resolution because it allows a sufficient number of points per peak (>12), signal intensity and good selectivity. Therefore, the choice of appropriate resolution is the key step for accurate qualitative analysis and quantitation The extracted ion chromatogram and mass spectrogram for monolinuronion ion (*m*/*z* 214.0504) spiked at 10 μg/kgin orange juice with ±1 mDa mass extraction window are represented in Figure 3. In this figure, at the resolution tested (15,000, 30,000 FWHM), there was a lower peakintensity with a higher mass error. However, when increasing the resolution to 60,000 and 120,000, the chromatographic peak could be observed in the same conditions, and the selectedion was now clearly separated into two differentiated peaks: one belonging to monolinuron and the other one belonging to a matrix interference(*m*/*z* 214.06683). Therefore, the optimum acquisition conditions in full scan mode (*m*/*z* 50–500) were a resolving power of 60,000. Therefore, this method can improve the credibility and accuracy of pesticide screening results in complex substrates.

### 3.4. Qualitative Screening and Confirmation

**Figure 4:**
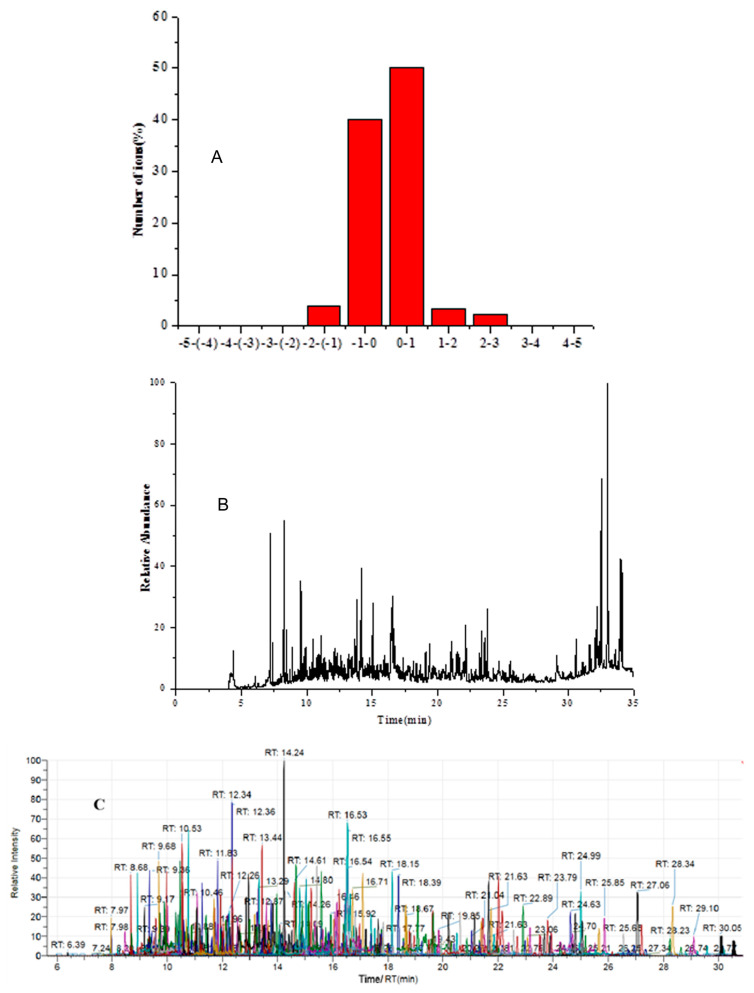
Mass error (**A**), total ion chromatogram (**B**) and multiple extracted ion chromatograms (**C**) of 350 of pesticides spiked in the orange juice matrix at 100 μg/kg.

For identification the requirements of SANTE/12682/2019 were followed, which means that the relative intensities or ratios of selective ions, expressed as a ratio relative to the most intense ion, which are used for identification, should match with the reference ion ratio [38]. For accurate mass measurement/high resolution mass spectrometry, the variability of ion ratios is not only affected by S/N of the peaks in the extracted ion chromatograms, but may also be affected by the way fragment ions are generated, and by the matrix. For this reason, no generic guidance value for ion ratio can be given. Due to the added value of accurate mass measurement, matching ion ratios are not necessary. However, they may provide additional support for identification. Besides, document also sets requirements with respect to retention time deviations (≤±0.1 min from reference retention time) and mass deviations (≤±5 ppm). All the 350 target compounds can be identified accurately by using the database established in this paper. Mass error, total ion chromatogram, and multiple extracted ion chromatograms of 350 pesticides spiked in the orange juice matrix at 100 μg/kg, as shown in Figure 4. The precision of mass deviations meets the requirement of 5 ppm, with most of them within ±1 ppm (Figure 4A). The total ion chromatogram (Figure 4B) contains rich chemical information, but the chromatographic peak of the target substance is not visible. From the extracted ion chromatograms (Figure 4C) of the orange juice matrix, 350 target compounds could be confirmed and quantified accurately.

### 3.5. Matrix Effect

Signal suppression or enhancement may occur when pesticides exist in sample matrix solution. The phenomenon took place when analyte interacted with active sites in the GC system such as the liner and column, and the occurrence of the matrix may have affected the ionization of analytes in the MS system. Suppression or enhancement of the analyte response can vary considerably from matrix to matrix and differ substantially in pure solvent and matrixes. To determine the matrix effects (ME), theslope s of the calibration curves obtained in matrix-matched standards were compared with those acquired from solvent-based standards. In the present study, the matrix effect was considered to be ignored if the slope ratios of matrix/solvent were inthe range of 0.9–1.1, while it would be regarded as matrix suppression effect if the value was lower than 0.9, and it would be taken as a matrix enhancement effect when the value was larger than 1.1. In this experiment, the matrix effects of eight typical matrices (orange juice, apple juice, grape juice, strawberry juice, celery juice, carrot juice, cucumber juice, tomato juice) were investigated. Matrix effects were evaluated by comparing the slope ratios matrix/solvent of 350 pesticide standards, prepared in fruits and vegetable juices blank extracts, with standards in solvent. As can be seen, most of the pesticides exhibited matrix enhancement effects. When m-PFC was used for clean-up of the selected matrices, a total of 21.1% and 17.1% pesticides could be deemed tohaveno matrix effects in the orange juice and celery juice matrix respectively, as shown in Supplementary Materials Table S2. As for the matrix suppression orenhancement effect, QuEChERS (simple matrix) was the strongest, while m-PFC was the weakest. This indicated that the cleanup effect of the m-PFC column was better than QuEChERS (simple matrix) and QuEChERS (complex matrix). When using m-PFC, minimalmatrixenhancement effects occurred on most compounds studied (slightly over 1.1) in the eight matrices. The results suggested that some enhancement of the MS signal had occurred with substances in extracts, which showed low but significant matrix effect. The results proved that the matrix-matched calibration standards are indispensable for accurate quantification by GC-Orbitrap/MS.

### 3.6. Validation of the Method

The accuracy and repeatability of the screening method were investigated under the fortified concentrations of 10 mg/kg, 100 mg/kg and 500 mg/kg in eight different matrices (orange juice, apple juice, grape juice, strawberry juice, celery juice, carrot juice, cucumber juice, tomato juice). A blank matrix standard solution was prepared, and the corresponding concentration (x-axis) was plotted by the peak area (y-axis). The concentrations of the multi-standard working solution were 0.005–0.5 μg/mL. And results on mass spectrometer demonstrated that the R-squared of 350 pesticides were no less than 0.990. All selected pesticides were detected in three extracts at 5 μg/kg level. The LODs and LOQs of the method were determined by the addition of blank samples. The LODs and LOQs were calculated as the concentration corresponding to the signals of 3 and 10 times the standard deviation of the baseline noise, respectively. The LODs and LOQs for the 350 pesticides were found to be 0.3–3 μg/kg and 1–10.0 μg/kg. Due to the reduced matrix interference, the sensitivity of the proposed method was significantly improved. Recovery and repeatability experiments were performed at three levels (10, 100, and 500 μg/kg) with six replicates at each level to evaluate the accuracy and precision of the methods. The accuracy was estimated by recoveries (%) and the precision was evaluated by RSDs (%) of the spiked samples. The results showed that the mean recoveries of 350compounds were 72.8–122.4% at three levels. Additionally, the average RSDs were 2.0–10.8%. The results fulfilled the requirements for pesticide residue analysis. The correlation coefficient, LODs, LOQs, MRLs, mean recoveries, and average RSDs of 350 compounds in the orange juice and celery juice matrix were detailed in Supplementary Materials Table S3. The results of the three kinds of cleanup methods were shown in Table 1. Recoveries and RSDs showed no significant differences between m-PFC and previous m-PFC. The recovery rate of the traditional QuEChERS was the worst. The LODs and LOQs were lower than the results in previous m-PFC and traditional QuEChERS. The number of pesticides in this work has reached 350, which was much more than the other two methods. Besides, each sample took less than two minutes for m-PFC cleanup.

### 3.7. Practical Screening

**Figure 5:**
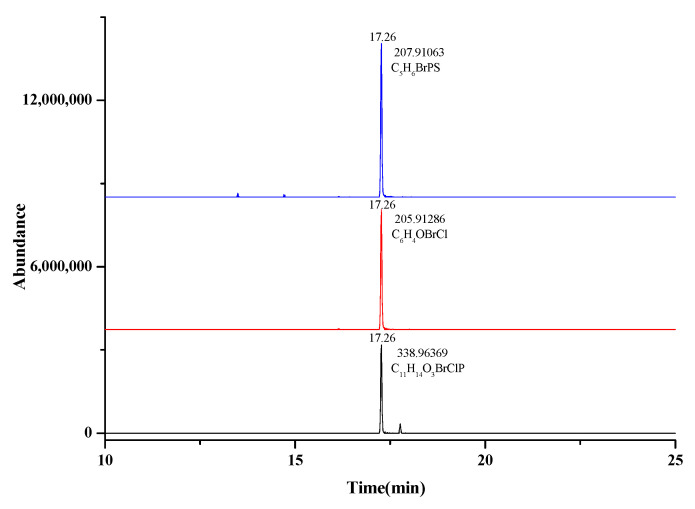
EIC corresponding to the fragment ions and abundance of profenofos in the orange juice sample.

The method was further applied in screening of 240 vegetable and fruit juice samples (orange juice, apple juice, grape juice, strawberry juice, celery juice, carrot juice, cucumber juice, tomato juice). There were 30 samples for each kind. The identified pesticides and range of residues in eight kinds of fruit and vegetable juice are shown in Table 2. All samples were extracted and cleaned up according to Section 2.4. For the compounds with concentrations out of the linear range, samples were re-diluted with the dilution factor of samples adjusted to ensure the concentration was quantitatively evaluated based on our linear range. The developed database was used for data retrieval. Quantification was conducted through the peak areas of the quantitative ion in identified samples. Based on the database and preset identification rules, 139 vegetable and fruit juice samples were screened as positive samples. Among all the detected pesticides, metalaxyl, chlorfenapyr, tebuconazole, dimethomorph, difenoconazole, pyraclostrobin, pyrimethanil, chlorpyrifos, propiconazole, procymidone had the highest detection rate. Meanwhile, orange juice, grape juice, strawberry juice, celery juice and cucumber juice had the highest detection rate in the 200 samples. MRLs of some pesticides in orange juice, tomato juice and grape juices have been establishedin our country [39]. For other fruit and vegetable juice, the corresponding MRLs were not specified. Neither the Codex Alimentarius Commission [40] nor the European Union [41] have given MRLs for pesticide residues in fruit and vegetable juices. Therefore, the formulation of relevant standards should be accelerated to provide reference for the MRLs of pesticides in fruit and vegetable juice. A good example of the performance of the GC-Orbitrap/MS was represented in Figure 5, where profenofos at 30.8 μg/kg was shown in the orange juice sample, with the overlaid extracted ion chromatogram for the three ions (*m*/*z* 338.96369, 205.91286, 207.91063), the correct retention time and ion ratio, compared to the theoretical value. The method proved to be suitable for the determination of 350 pesticide residues in fruit and vegetable juices, and it also provided targeting and pertinence for the determination of pesticide residues.

### 3.8. Retrospective Evaluation

The method was successfully applied for a pesticides risk assessment of vegetable and fruit juice samples. The detection mode was established on potential and known pesticides, where their chromatographic and mass spectrometric information were examined and collected. However, unknown and non-target risk compounds were ignored. GC-Orbitrap/MS used a full-scan mode to capture precise mass numbers, allowing for more comprehensive data collection. Data collection was independent of the number of compounds in the database. Therefore, the data could be reviewed and reanalyzed after the acquisition to find and identify unknown peaks and expand the target range. For example, dibutyl phosphate and tributyl phosphate are new compounds that were added to 350 databases and verified by actual samples. The results showed that dibutyl phosphate was detected in three grape juice samples in the range of 15–45 μg/kg. This method can be used to expand and analyze the target compounds without collecting data again. Further work will be focused on non-target screening for recognizing and monitoring risk factors. 

## 4. Conclusions

In this work, a methodology intended for vegetable and fruit juice samples assessment based on the combination of m-PFC cleanup and GC-Orbitrap/MS was successfully developed. Taking advantage of the high sensitivity of the Orbitrap analyzer when operating in full scan mode, and the valuable accurate mass information provided, retrospective analyses (post-target and non-target screenings), in addition to target analyses were carried out. GC-Orbitrap/MS was suited to the quantitative determination and identification of pesticides in vegetable and fruit juice samples using matched matrixes. These good results present the advantages derived from full scan analysis applicable to other compounds not present in the selected and retrospective evaluation together with easier scope management compared with GC-MS/MS. The m-PFC purification column could not only effectively remove pigments, organic acids, fat and water, but also save sample preparation time and avoid the losses caused by solvent transfers. The developed cleanup procedure did not need additional vortex or centrifugation steps. Compared to traditional QuEChERS, m-PFC made this method labor-saving and easy, as well as robust and reproducible. The target method was fully validated for the determination of 350 pesticides in vegetable and fruit juice samples providing excellent linearity, sensitivity, trueness (relative recovery values ranged from 73.2 to 122.4%) and precision (RSD < 10.8%) for all target compounds. An in-house database containing close to 350 pesticides was used in the post-target screening, and a restrictive workflow was proposed for the identification of non-target ones. The overall outcome of the evaluation is that GC-Orbitrap/MS is considered highly suited for pesticide residue analysis. An evaluation of the screening capabilities is the subject of current research. This method can be used as a fast, reliable, efficient and practical tool for the pesticides at trace levels for various vegetable and fruit juices, which saves more time and expenses.

## Figures and Tables

**Table 1:** Comparison of the proposed method with other methods.

Method	Recoveries (%)	RSD (%)	LOD (μg/kg)	LOQ (μg/kg)	Number of Pesticides	Cleanup Time Cost per Sample (Min)
m-PFC	72–122	<10.8	0.3–3.0	1.0–10.0	350	<2
previous m-PFC	70–125	<15.0	1.0–3.0	3.0–10.0	<200	2–3
traditional QuEChERS	60–125	<15.0	1.0–3.0	3.0–10.0	250	5

**Table 2:** Pesticide residues in real vegetable and fruit juice samples.

Samples	Pesticide	Positive Samples		Range of Residues (μg/kg)
		N	%	
orange juice	Propiconazole	8	26.7	41.2–200.4
	Trifloxystrobin	14	46.7	20.8–300.7
	Chlorpyrifos	12	40.0	10.5–100.9
	Imazalil	13	43.3	30.4–425.9
	Pyraclostrobin	11	36.7	30.1–224.5
	Malathion	2	6.7	30.6–230.4
	Pyrimethanil	7	23.3	20.2–100.7
	profenofos	14	46.7	40.2–600.4
apple juice	Difenoconazole	8	26.7	21.2–420.4
	Chlorpyrifos	6	20.0	36.8–300.7
	Flusilazole	3	10.0	50.5–80.9
	Tebuconazole	15	50.0	10.1–344.1
	Dimethomorph	7	23.3	30.8–250.2
grape juice	Bifenthrin	4	13.3	20.1–200.3
	Difenoconazole	6	20.0	20.2–600.4
	Dimethomorph	5	16.7	50.2–520.4
	Propiconazole	8	26.7	16.2–312.7
	Pyraclostrobin	12	40.0	25.5–400.9
	Pyrimethanil	7	23.3	12.4–465.3
	Spirodiclofen	9	30.0	10.1–344.1
	Metalaxyl	16	53.3	31.8–750.5
	Chlorfenapyr	13	43.3	40.1–500.3
	Tebuconazole	10	33.3	15.2–400.4
strawberry juice	Cyhalothrin	6	20.0	20.1–100.3
	Dichlorvos	4	13.3	10.2–62.4
celery juice	Chlorfenapyr	16	53.3	43.2–520.4
	Difenoconazole	12	40.0	12.2–344.7
	Metalaxyl	13	43.3	20.5–450.9
	Prochloraz	12	40.0	43.4–805.3
	Propiconazole	11	36.7	30.1–305.1
	Tebuconazole	8	26.7	31.8–750.5
	Isoprocarb	3	10.0	42.1–80.3
	Dimethomorph	11	36.7	15.2–520.4
	Pyraclostrobin	12	40.0	21.1–430.3
	Oxadixyl	7	23.3	18.2–90.4
	Phorate	4	13.3	10.2–400.4
	Metalaxyl	15	50.0	12.2–343.7
	Pendimethalin	9	30.0	21.5–320.6
carrot juice	Triadimefon	6	20.0	12.2–505.3
	Phorate	3	10.0	16.1–134.3
cucumber juice	Metalaxyl	14	46.7	31.2–850.5
	Procymidone	11	36.7	20.1–440.3
	Pyrimethanil	8	26.7	15.2–330.4
	Chlorpyrifos	10	33.3	22.1–426.3
	Dimethoate	5	16.7	20.2–560.4
	Fluopyram	4	13.3	20.2–800.4
	Omethoate	6	20.0	16.2–122.4
	Dimethomorph	10	33.3	18.5–423.9
	Endosulfan	3	10.0	12.4–225.3
	Chlorfenapyr	13	43.3	12.1–444.1
	Pyridaben	12	40.0	31.8–651.5
tomato juice	Chlorfenapyr	13	43.3	38.1–623.3
	Difenoconazole	10	33.3	15.2–442.4
	Pyrimethanil	9	30.0	20.1–330.3
	Dimethomorph	11	36.7	16.3–422.1
	Procymidone	12	40.0	22.4–445.3
	Tebuconazole	14	46.7	10.1–384.1

## Supplementary Materials

The following are available online at https://www.mdpi.com/article/10.3390/foods10071651/s1, Table S1: Retention time, fragment ions and accurate mass of 350 pesticides, Table S2: Matrix effect (ME) obtained for the target compounds in the orange juice and celery juice matrix, Table S3: Correlation coefficients (R^2^), limits of detection, spiked recoveries and RSDs of the 350 pesticides in orange juice and celery juice (*n* = 6).
