# Supplementary material for: Rapid Screening of 350 Pesticide Residues in Vegetable and Fruit Juices by Multi-Plug Filtration Cleanup Method Combined with Gas Chromatography-Electrostatic Field Orbitrap High Resolution Mass Spectrometry
Source: Foods. 2021 Jul 16;10(7):1651. doi: 10.3390/foods10071651 (PMC8305287; doi:10.3390/foods10071651)
Supplement: Supplementary file 1 [file foods-10-01651-s001.zip › foods-1233980-supplementary.pdf]

Table S1. Retention time, fragment ions and accurate mass of 350 pesticides.

Pesticides	CAS	Molecular Formula	Retention time (min)	Quantitative ion(m/z)	Qualitative ion(m/z)	
					1	2
Clopyralid	1702-17-6	$C_6H_3Cl_2NO_2$	6.37	146.96371	76.01817	111.99485
Dichlorvos	62-73-7	$C_4H_7O_4PCl_2$	7.98	184.97650	186.97355	144.98158
Methamidophos	10265-92-6	$C_2H_8NO_2PS$	8.01	141.00079	112.01585	125.97731
Thiofanox	39196-18-4	$C_9H_{18}N_2O_2S$	8.19	115.09917	161.08689	83.07295
Allidochlor	93-71-0	$C_8H_{12}NOCl$	8.26	138.09134	132.02107	96.08078
Dichlobenil	1194-65-6	$C_7H_3Cl_2N$	8.67	170.96371	100.01818	172.96075
EPTC	759-94-4	$C_9H_{19}NOS$	8.70	128.10699	132.08415	160.07906
Dichlormid	37764-25-3	$C_8H_{11}Cl_2NO$	8.71	172.05236	108.08077	165.98209
2,4,6-Trichlorophenol	88-06-2	$C_6H_3OCl_3$	8.72	195.92440	199.91850	197.92145
3,5-Dichloroaniline	626-43-7	$C_6H_5Cl_2N$	9.04	160.97936	162.97640	126.01050
O-Phthalimide	85-41-6	$C_8H_5NO_2$	9.14	147.03147	103.04165	104.02566
Mevinphos	7786-34-7	$C_7H_{13}O_6P$	9.17	192.01821	164.02330	127.01547
Acephate	30560-19-1	$C_4H_{10}NO_3PS$	9.24	136.01581	112.01581	94.00524
Vernolate	1929-77-7	$C_{10}H_{21}NOS$	9.29	86.06004	161.08688	146.09979
Propham	122-42-9	$C_{10}H_{13}NO_2$	9.36	137.04712	179.09408	120.08077
Etridiazole	2593-15-9	$C_5H_5N_2OSCl_3$	9.39	210.94942	212.94646	182.91812
Pebulate	1114-71-2	$C_{10}H_{21}NOS$	9.39	128.10699	72.04439	161.08689
cis-1,2,3,6-Tetrahydrophthalimide	1469-48-3	$C_8H_9NO_2$	9.56	151.06280	123.06790	122.06000
Chloroneb	2675-77-6	$C_8H_8O_2Cl_2$	9.76	190.96611	192.96316	205.98958
Tebuthiuron	34014-18-1	$C_9H_{16}N_4OS$	9.85	156.05899	89.01679	171.08246
Fenobucarb	3766-81-2	$C_{12}H_{17}NO_2$	9.92	121.06479	91.05423	93.06988
Pentachlorobenzene	608-93-5	C_6HCl_5	9.94	247.85154	251.84563	249.84858
Isoprocarb	2631-40-5	$C_{11}H_{15}NO_2$	9.97	121.06479	136.08827	103.05423
Molinate	2212-67-1	$C_9H_{17}NOS$	10.00	126.09134	187.10254	98.09643
Heptenophos	23560-59-0	$C_9H_{12}CLO_4P$	10.29	124.00743	215.04677	200.02330
Chlorfenprop-methyl	14437-17-3	$C_{10}H_{10}O_2Cl_2$	10.42	165.01016	196.02855	167.00721
Omethoate	1113-02-6	$C_5H_{12}NO_4PS$	10.46	156.00045	110.01273	140.97697
Propoxur	114-26-1	$C_{11}H_{15}NO_3$	10.56	110.03623	82.04131	152.08318
Tecnazene	117-18-0	$C_6HNO_2CL_4$	10.58	200.88269	177.91383	260.87264
Propachlor	1918-16-7	$C_{11}H_{14}NOCl$	10.60	120.08077	176.10699	169.02889
Diphenylamine	122-39-4	$C_{12}H_{11}N$	10.69	169.08860	167.07295	168.08078
Ethoprophos	13194-48-4	$C_8H_{19}O_2PS_2$	10.76	157.96196	199.00110	200.00891
Cycloate	1134-23-2	$C_{11}H_{21}NOS$	10.78	154.12264	155.12600	72.04439
2,3,5,6-tetrachloroaniline	3481-20-7	$C_6H_3Cl_4N$	10.79	230.89846	232.89551	157.95588
Atrazine-desethyl	6190-65-4	$C_6H_{10}N_5Cl$	10.99	172.03844	145.01497	187.06192
Dicrotophos	141-66-2	$C_8H_{16}NO_5P$	11.06	127.01547	193.02604	111.06787
Methabenzthiazuron	18691-97-9	$C_{10}H_{11}N_3OS$	11.06	136.02154	164.04027	135.01372
Trifluralin	1582-09-8	$C_{13}H_{16}N_3O_4F_3$	11.07	264.02152	306.06961	248.02775

Bendiocarb	22781-23-3	$C_{11}H_{13}NO_4$	11.08	151.03897	126.03114	223.08390
Benfluralin	1861-40-1	$C_{13}H_{16}F_3N_3O_4$	11.11	292.05282	276.05905	318.10600
Sulfotep	3689-24-5	$C_8H_{20}O_5P_2S_2$	11.19	322.02218	209.89698	173.95687
Cadusafos	95465-99-9	$C_{10}H_{23}O_2PS_2$	11.24	158.96979	130.93848	213.01673
Tebutam	35256-85-0	$C_{15}H_{23}NO$	11.26	91.05422	190.12264	233.17741
Promecarb	2631-37-0	$C_{12}H_{17}NO_2$	11.26	135.08044	107.08552	150.10391
Phorate	298-02-2	$C_7H_{17}O_2PS_3$	11.33	75.02629	230.97315	260.01227
Atratone	1610-17-9	$C_9H_{17}N_5O$	11.60	169.09581	154.07233	211.14276
3,4,5-Trimethacarb	2686-99-9	$C_{11}H_{15}NO_2$	11.68	121.06479	91.05422	136.08826
Dicloran	99-30-9	$C_6H_4Cl_2N_2O_2$	11.69	175.96644	159.97153	207.96148
Pentachloroanisole	1825-21-4	$C_7H_3Cl_5O$	11.71	264.83567	238.83781	279.85915
Ethoxyquin	91-53-2	$C_{14}H_{19}NO$	11.71	202.12264	174.09134	203.12599
Prometon	1610-18-0	$C_{10}H_{19}N_5O$	11.72	168.08798	210.13493	225.15841
Atrazine	1912-24-9	$C_8H_{14}ClN_5$	11.83	200.06975	202.06680	173.04627
Monolinuron	1746-81-2	$C_9H_{11}ClN_2O_2$	11.88	126.01050	152.99759	214.05036
Propazine	139-40-2	$C_9H_{16}N_5Cl$	11.91	214.08539	187.06192	229.10887
Clomazone	81777-89-1	$C_{12}H_{14}ClNO_2$	11.93	204.10190	89.03857	125.01525
α-Hexachlorocyclohexane	319-84-6	$C_6H_6Cl_6$	11.94	180.93730	145.96845	218.91103
Terbumeton	33693-04-8	$C_{10}H_{19}N_5O$	11.94	210.13493	169.09581	225.15841
β-Hexachlorocyclohexane	319-85-7	$C_6H_6Cl_6$	11.98	180.93730	145.96845	218.91103
Aminocarb	2032-59-9	$C_{11}H_{16}N_2O_2$	12.03	151.09916	136.07569	150.09134
Isocarbamid	30979-48-7	$C_8H_{15}N_3O_2$	12.04	142.06110	130.06110	113.03455
Cyromazine	66215-27-8	$C_6H_{10}N_6$	12.05	151.07267	165.08832	166.09614
γ-Hexachlorocyclohexane	58-89-9	$C_6H_6Cl_6$	12.11	180.93730	145.96845	218.91103
Propetamphos	31218-83-4	$C_{10}H_{20}NO_4PS$	12.11	138.01369	193.97971	222.03482
Cycluron	2163-69-1	$C_{11}H_{22}N_2O$	12.12	198.17266	127.08659	169.13354
Terbuthylazine	5915-41-3	$C_9H_{16}N_5Cl$	12.13	186.05409	188.05114	201.07757
Terbufos	13071-79-9	$C_9H_{21}O_2PS_3$	12.15	230.97315	174.91055	202.94185
Cyanophos	2636-26-2	$C_9H_{10}NO_3PS$	12.16	243.01135	124.98206	109.00491
Trietazine	1912-26-1	$C_9H_{16}N_5Cl$	12.16	200.06974	214.08539	229.10887
Quintozene	82-68-8	$C_6NO_2Cl_5$	12.22	213.87191	248.84076	294.83366
Fonofos	944-22-9	$C_{10}H_{15}OPS_2$	12.26	246.02964	137.01845	108.98715
Pyroquilon	57369-32-1	$C_{11}H_{11}NO$	12.27	173.08351	144.08077	172.07569
Dinoterb	1420-07-1	$C_{10}H_{12}N_2O_5$	12.34	225.05059	177.04204	161.04712
Pyrimethanil	53112-28-0	$C_{12}H_{13}N_3$	12.34	198.10257	199.11039	183.07909
Diazinon	333-41-5	$C_{12}H_{21}N_2O_3PS$	12.35	179.11789	199.06309	304.10050
Flufenoxuron	101463-69-8	$C_{21}H_{11}CLF_6N_2O_3$	12.45	331.00177	268.03800	296.03292
Disulfoton	298-04-4	$C_8H_{19}O_2PS_3$	12.50	88.03412	153.01336	141.96704
Paraoxon-methyl	950-35-6	$C_8H_{10}NO_6P$	12.51	230.02129	247.02402	200.02330
Secbumeton	26259-45-0	$C_{10}H_{19}N_5O$	12.51	196.11928	169.09581	210.13493
Aziprotryne	4658-28-0	$C_7H_{11}N_7S$	12.52	182.04949	139.00729	225.07911
Dinitramine	29091-05-2	$C_{11}H_{13}F_3N_4O_4$	12.52	305.08560	244.05665	261.05939
Fenfuram	24691-80-3	$C_{12}H_{11}NO_2$	12.56	201.07843	184.05188	109.02841
δ-Hexachlorocyclohexane	319-86-8	$C_6H_6Cl_6$	12.60	180.93730	145.96845	218.91103

Mexacarbate	315-18-4	$C_{12}H_{18}N_2O_2$	12.62	165.11481	164.10699	222.13627
Isazofos	42509-80-8	$C_7H_{13}N_3O_3PSCl$	12.65	162.04268	161.03504	177.01202
Chlorothalonil	1897-45-6	$C_8Cl_4N_2$	12.70	263.88101	193.94330	228.91215
Triallate	2303-17-5	$C_{10}H_{16}CL_3NOS$	12.70	268.03242	270.02947	142.92166
Tebupirimfos	96182-53-5	$C_{13}H_{23}N_2O_3PS$	12.81	234.02225	261.04572	276.06920
musk ambrette	83-66-9	$C_{12}H_{16}N_2O_5$	12.82	253.08189	251.10263	268.10537
Oxabetrinil	74782-23-3	$C_{12}H_{12}N_2O_3$	12.84	73.02840	103.04165	114.03382
Iprobenfos	26087-47-8	$C_{13}H_{21}O_3PS$	12.86	204.00045	171.02056	246.04740
Fluroxypyr	69377-81-7	$C_7H_5N_2O_3FCl_2$	12.91	180.97300	208.96792	195.96009
Pirimicarb	23103-98-2	$C_{11}H_{18}N_4O_2$	12.94	238.14243	166.09749	137.07094
Monalide	7287-36-7	$C_{13}H_{18}NOCl$	12.94	197.06019	127.01832	239.10714
Furmecyclox	60568-05-0	$C_{14}H_{21}NO_3$	12.97	123.04406	251.15159	124.04741
Benoxacor	98730-04-2	$C_{11}H_{11}NO_2Cl_2$	12.98	120.04439	259.01613	261.01318
Pentachloroaniline	527-20-8	$C_6H_2Cl_5N$	13.13	262.86244	191.91690	229.89063
Benfuresate	68505-69-1	$C_{12}H_{16}O_4S$	13.16	163.07536	121.06479	256.07638
Dioxacarb	6988-21-2	$C_{11}H_{13}NO_4$	13.16	121.02840	165.05462	166.06244
Cyprazine	22936-86-3	$C_9H_{14}N_5Cl$	13.23	212.06974	170.02279	226.08539
Phosphamidon	13171-21-6	$C_{10}H_{19}NO_5PCl$	13.24	138.09134	193.02603	264.09953
Dichlorprop	120-36-5	$C_9H_8O_3Cl_2$	13.25	161.96337	132.96093	188.98684
Dichlofenthion	97-17-6	$C_{10}H_{13}CL_2O_3PS$	13.25	222.93818	250.96948	279.00060
Fenthion	55-38-9	$C_{10}H_{15}O_3PS_2$	13.25	222.93818	250.96948	279.00060
Propanil	709-98-8	$C_9H_9NOCl_2$	13.25	160.97935	162.97640	219.00262
2,4-DB	94-82-6	$C_{10}H_{10}O_3Cl_2$	13.25	161.96337	125.98669	97.99177
Chlorthiamid	1918-13-4	$C_7H_5Cl_2NS$	13.28	169.98257	171.97962	204.95142
Dimethachlor	50563-36-5	$C_{13}H_{18}NO_2Cl$	13.28	197.06019	148.07569	134.09643
Metribuzin	21087-64-9	$C_8H_{14}N_4OS$	13.30	198.06955	144.04641	182.03825
Dimethenamid	87674-68-8	$C_{12}H_{18}NO_2SCl$	13.31	154.06849	230.04008	232.03713
Bromobutide	74712-19-9	$C_{15}H_{22}NOBr$	13.34	119.08552	120.08077	232.16959
Terbucarb	1918-11-2	$C_{17}H_{27}NO_2$	13.43	205.15869	177.12739	220.18216
Malaoxon	1634-78-2	$C_{10}H_{19}O_7PS$	13.45	268.01650	194.98754	238.97737
Vinclozolin	50471-44-8	$C_{12}H_9Cl_2NO_3$	13.47	178.04180	212.00283	284.99539
Parathion-methyl	298-00-0	$C_8H_{10}NO_5PS$	13.48	263.00118	124.98210	245.99844
Chlorpyrifos-methyl	5598-13-0	$C_7H_7Cl_3NO_3PS$	13.49	285.92558	287.92263	289.91968
Transfluthrin	118712-89-3	$C_{15}H_{12}O_2F_4Cl_2$	13.53	163.01653	127.03090	335.04564
Simetryn	1014-70-6	$C_8H_{15}N_5S$	13.55	213.10426	155.03859	170.04949
Fuberidazole	3878-19-1	$C_{11}H_8N_2O$	13.56	184.06311	156.06820	183.05529
Tolclofos-methyl	57018-04-9	$C_9H_{11}O_3PSCL_2$	13.60	264.98496	249.96166	266.98200
Alachlor	15972-60-8	$C_{14}H_{20}NO_2Cl$	13.66	188.10699	202.12260	160.11208
Ametryn	834-12-8	$C_9H_{17}N_5S$	13.66	227.11991	170.04949	185.07296
Heptachlor	76-44-8	$C_{10}H_5CL_7$	13.71	269.81257	100.00742	336.84874
Prometryn	7287-19-6	$C_{10}H_{19}N_5S$	13.74	241.13557	184.06514	199.08862
Acetochlor	34256-82-1	$C_{14}H_{20}NO_2Cl$	13.75	223.07584	162.09134	174.09134
Paraoxon-ethyl	311-45-5	$C_{10}H_{14}NO_6P$	13.76	275.05533	247.02403	139.05186
Metalaxyl	57837-19-1	$C_{15}H_{21}NO_4$	13.77	160.11207	206.11755	146.09642
Tridiphane	58138-08-2	$C_{10}H_7OCl_5$	13.83	186.97119	172.95554	284.92160

Octachlorodipropyl ether	127-90-2	$C_6H_6OCl_8$	13.88	129.91383	108.96063	142.92165
Prosulfocarb	52888-80-9	$C_{14}H_{21}NOS$	13.88	128.10699	86.06004	251.13383
Fenpropidin	67306-00-7	$C_{19}H_{31}N$	13.96	98.09643	273.24510	258.22160
1-Naphthylacetamide	86-86-2	$C_{12}H_{11}NO$	14.04	141.06987	142.07770	185.08351
Dithiopyr	97886-45-8	$C_{15}H_{16}NO_2F_5S_2$	14.04	306.05479	258.05314	354.05816
Orbencarb	34622-58-7	$C_{12}H_{16}NOSCl$	14.07	222.09471	125.01525	100.07569
Terbutryn	886-50-0	$C_{10}H_{19}N_5S$	14.07	226.11209	185.07297	170.04949
Spiroxamine	118134-30-8	$C_{18}H_{35}NO_2$	14.10	100.11207	126.12772	198.14885
Methiocarb	2032-65-7	$C_{11}H_{15}NO_2S$	14.14	168.06034	153.03686	154.04022
Fenitrothion	122-14-5	$C_9H_{12}NO_5PS$	14.15	260.01409	124.98206	277.01683
Pirimiphos-methyl	29232-93-7	$C_{11}H_{20}N_3O_3PS$	14.18	290.07227	276.05662	305.09575
Methiocarb sulfone	2179-25-1	$C_{11}H_{15}NO_4S$	14.21	200.05017	197.02669	197.02669
Ethofumesate	26225-79-6	$C_{13}H_{18}O_5S$	14.21	207.10157	161.05971	179.07027
Linuron	330-55-2	$C_9H_{10}N_2O_2Cl_2$	14.26	159.97153	61.05221	248.01138
Probenazole	27605-76-1	$C_{10}H_9NO_3S$	14.32	130.06519	103.04165	158.06004
Noruron	18530-56-8	$C_{13}H_{22}N_2O$	14.33	153.10224	193.13353	207.14919
Quinoclamine	2797-51-5	$C_{10}H_6NO_2Cl$	14.36	172.03930	144.04439	207.00815
Dipropetryn	4147-51-7	$C_{11}H_{21}N_5S$	14.37	255.15121	222.17132	184.06514
Malathion	121-75-5	$C_{10}H_{19}O_6PS_2$	14.40	124.98206	99.00767	173.08083
Thiobencarb	28249-77-6	$C_{12}H_{16}ClNOS$	14.43	257.06356	100.07569	125.01525
Diethofencarb	87130-20-9	$C_{14}H_{21}NO_4$	14.50	267.14651	225.09956	168.02913
Phorate sulfoxide	2588-03-6	$C_7H_{17}O_3PS_3$	14.56	124.92791	170.96978	199.00108
Metolachlor	51218-45-2	$C_{15}H_{22}NO_2Cl$	14.60	162.12772	211.07584	238.09931
Fenpropimorph	67564-91-4	$C_{20}H_{33}NO$	14.66	128.10699	110.09642	173.13247
Cyanazine	21725-46-2	$C_9H_{13}ClN_6$	14.68	225.06500	212.05717	240.08847
Chlorpyrifos	2921-88-2	$C_9H_{11}CL_3NO_3PS$	14.70	196.91964	257.89428	313.95688
Parathion	56-38-2	$C_{10}H_{14}NO_5PS$	14.72	291.03248	155.00355	185.99510
Flufenacet	142459-58-3	$C_{14}H_{13}N_3O_2F_4S$	14.78	210.97836	136.05571	151.07918
Rabenzazol	40341-04-6	$C_{12}H_{12}N_4$	14.78	212.10564	170.07127	195.07909
4,4'-Dichlorobenzophenone	90-98-2	C13H8Cl2O	14.79	138.99451	110.99960	249.99467
Triadimefon	43121-43-3	$C_{14}H_{16}ClN_3O_2$	14.79	208.02722	210.02426	181.01632
Chlorthal-dimethyl	1861-32-1	$C_{10}H_6CL_4O_4$	14.85	300.88013	298.88308	331.89852
Dicapthon	2463-84-5	$C_8H_9NO_5PSCl$	14.85	261.99335	124.98206	216.00045
Isofenphos-oxon	31120-85-1	$C_{15}H_{24}NO_5P$	14.86	200.99473	229.02603	272.06823
Isocarbophos	24353-61-5	$C_{11}H_{16}NO_4PS$	14.89	135.99774	230.00353	121.02841
Tetraconazole	112281-77-3	$C_{13}H_{11}Cl_2F_4N_3O$	14.91	336.05212	136.00742	170.97608
Isobenzan	297-78-9	$C_9H_4CL_8O$	14.99	407.77649	274.85641	310.83309
Flurochloridone	61213-25-0	$C_{12}H_{10}Cl_2F_3NO$	15.00	174.05251	311.00860	313.00565
Fenson	80-38-6	$C_{12}H_9O_3SCl$	15.02	267.99554	141.00048	269.99260
Pyracarbolid	24691-76-7	$C_{13}H_{15}NO_2$	15.12	125.05970	217.10973	97.02840
Dodemorph	1593-77-7	$C_{18}H_{35}NO$	15.12	154.12264	238.21654	281.27132
Mgk 264	113-48-4	$C_{17}H_{25}NO_2$	15.13，15.44	164.07060	209.14103	210.14886
Butralin	33629-47-9	$C_{14}H_{21}N_3O_4$	15.15	266.11353	236.10297	220.10805
Carbaryl	63-25-2	$C_{12}H_{11}NO_2$	15.15	144.05696	115.05422	116.06205

Diphenamid	957-51-7	$C_{16}H_{17}NO$	15.20	167.08552	165.06987	152.06205
Pirimiphos-ethyl	23505-41-1	$C_{13}H_{24}N_3O_3PS$	15.28	168.05899	318.10357	333.12705
Isodrin	465-73-6	$C_{12}H_8Cl_6$	15.35	192.93731	361.87520	194.93436
Aldrin	309-00-2	$C_{12}H_8CL_6$	15.35	260.85936	290.92963	326.90631
Isopropalin	33820-53-0	$C_{15}H_{23}N_3O_4$	15.35	280.12918	238.08223	264.13426
Cyprodinil	121552-61-2	$C_{14}H_{15}N_3$	15.40	224.11822	225.12604	208.08692
Isofenphos-methyl	99675-03-3	$C_{14}H_{22}NO_4PS$	15.41	199.01547	230.98754	241.06242
Octachlorostyrene	29082-74-4	C_8Cl_8	15.53	305.81257	270.84371	379.74437
Metazachlor	67129-08-2	$C_{14}H_{16}ClN_3O$	15.55	209.06019	133.08860	211.05724
Dimethametryn	22936-75-0	$C_{11}H_{21}N_5S$	15.57	212.09644	185.07296	240.12774
Pendimethalin	40487-42-1	$C_{13}H_{19}N_3O_4$	15.59	252.09788	191.06893	162.07876
Disulfoton-sulfone	2497-6-5	$C_8H_{19}O_4PS_3$	15.61	153.01336	124.98206	213.01673
Phorate sulfone	2588-04-7	$C_7H_{17}O_4PS_3$	15.61	199.00108	124.98206	170.96978
Terbufos sulfone	56070-16-7	$C_9H_{21}O_4PS_3$	15.61	153.01336	199.00108	263.97081
Paclobutrazol	76738-62-0	$C_{15}H_{20}ClN_3O$	15.63	236.05851	138.02307	167.02581
Penconazole	66246-88-6	$C_{13}H_{15}N_3Cl_2$	15.63	248.09490	160.97333	158.97628
Chlozolinate	84332-86-5	$C_{13}H_{11}NO_5Cl_2$	15.71	186.95862	260.97680	188.95567
Pyrifenox	88283-41-4	$C_{14}H_{12}N_2OCl_2$	15.71	262.00590	186.95862	227.03705
Tolylfluanid	731-27-1	$C_{10}H_{13}N_2O_2FS_2Cl_2$	15.74	237.96548	181.07940	239.96253
Fosthiazate	98886-44-3	$C_9H_{18}NO_3PS_2$	15.79	195.01135	166.02119	226.98342
Phosfolan	947-02-4	$C_7H_{14}NO_3PS_2$	15.79	139.95658	167.98787	266.98342
Allethrin	584-79-2	$C_{19}H_{26}O_3$	15.83	123.11683	91.05423	136.08827
Isofenphos	25311-71-1	$C_{15}H_{24}NO_4PS$	15.83	213.03112	184.99982	216.97138
Captan	133-06-2	$C_9H_8NO_2SCl_3$	15.84	149.04713	105.03349	116.90601
Fipronil	120068-37-3	$C_{12}H_4Cl_2F_6N_4OS$	15.87	366.94294	368.93975	212.94801
Diclocymet	139920-32-4	$C_{15}H_{18}N_2OCl_2$	15.89，16.37	221.04761	172.99193	277.11021
Quinalphos	13593-03-8	$C_{12}H_{15}N_2O_3PS$	15.91	146.04746	157.07602	173.07094
Phenthoate	2597-03-7	$C_{12}H_{17}O_4PS_2$	15.92	273.98817	121.01064	245.99325
Triadimenol	55219-65-3	$C_{14}H_{18}N_3O_2Cl$	15.93	168.11314	112.05054	169.11649
Dinobuton	973-21-7	$C_{14}H_{18}N_2O_7$	15.99	211.03495	163.02639	205.06077
Furalaxyl	57646-30-7	$C_{17}H_{19}NO_4$	16.02	242.11756	152.07060	146.09643
Crotoxyphos	7700-17-6	$C_{14}H_{19}O_6P$	16.05	193.02604	127.01547	105.06988
Procymidone	32809-16-8	$C_{13}H_{11}NO_2Cl_2$	16.10	283.01613	96.05696	255.02122
Chlorbenside	103-17-3	$C_{13}H_{10}SCl_2$	16.14	125.01525	127.0123	267.98747
Chlorflurenol-methyl	2536-31-4	$C_{15}H_{11}O_3Cl$	16.21	215.02581	152.06205	274.03922
Chlordane	5103-71-9	$C_{10}H_6Cl_8$	16.31，16.57	372.82542	376.81952	374.82247
Methidathion	950-37-8	$C_6H_{11}N_2O_4PS_3$	16.32	145.00662	85.03964	147.00240
Haloxyfop-methyl	69806-40-2	$C_{16}H_{13}ClF_3NO_4$	16.38	375.04797	288.00288	179.98224
Bromophos-ethyl	4824-78-6	$C_{10}H_{12}O_3PSCl_2Br$	16.40	300.84869	241.87159	358.90906
Procyazine	32889-48-8	$C_{10}H_{13}N_6Cl$	16.42	210.05409	212.05114	252.08847
Disulfoton-sulfoxide	2497-07-6	$C_8H_{19}O_3PS_3$	16.60	183.97761	124.98206	167.98269
Tetrachlorvinphos	22248-79-9	$C_{10}H_9O_4PCl_4$	16.62	328.92985	203.92948	239.88756
Endosulfan	959-98-8	$C_9H_6O_3SCl_6$	16.66，18.36	236.84076	169.96846	159.98411
Mepanipyrim	110235-47-7	$C_{14}H_{13}N_3$	16.68	222.10257	221.09475	223.11040

Butachlor	23184-66-9	$C_{17}H_{26}NO_2Cl$	16.73	176.10699	188.10699	160.11208
Ditalimfos	5131-24-8	$C_{12}H_{14}NO_4PS$	16.80	242.97497	208.96949	271.00627
TCMTB	21564-17-0	$C_9H_6N_2S_3$	16.86	179.99361	166.98579	237.96876
trans-Nonachlor	39765-80-5	$C_{10}H_5Cl_9$	16.89	408.78350	404.78940	271.80962
Chlorfenson	80-33-1	$C_{12}H_8CL_2O_3S$	16.93	174.96150	176.95855	301.95657
Fenamiphos	22224-92-6	$C_{13}H_{22}NO_3PS$	16.94	303.10525	260.05047	217.00827
Picoxystrobin	117428-22-5	$C_{18}H_{16}NO_4F_3$	16.95	303.05016	173.05970	335.07637
Napropamide	15299-99-7	$C_{17}H_{21}NO_2$	16.99	271.15668	72.08078	115.05423
Hexaconazole	79983-71-4	$C_{14}H_{17}Cl_2N_3O$	17.05	213.99333	231.03380	174.97120
Flutolanil	66332-96-5	$C_{17}H_{16}NO_2F_3$	17.06	173.02087	281.06581	323.11276
Prothiophos	34643-46-4	$C_{11}H_{15}O_2PS_2Cl_2$	17.17	308.99341	238.91516	266.94664
Isoprothiolane	50512-35-1	$C_{12}H_{18}O_4S_2$	17.18	117.99054	161.98037	290.06410
Profenofos	41198-08-7	$C_{11}H_{15}BrClO_3PS$	17.25	338.96369	205.91286	207.91063
tricyclazole	41814-78-2	$C_9H_7N_3S$	17.34	189.03552	135.01372	161.01680
Pretilachlor	51218-49-6	$C_{17}H_{26}NO_2Cl$	17.35	162.12772	202.12264	238.09931
Dieldrin	60-57-1	$C_{12}H_8CL_6O$	17.43	262.85641	81.03349	260.85936
Oxadiazon	19666-30-9	$C_{15}H_{18}N_2O_3Cl_2$	17.50	174.95862	302.02194	344.06889
Iprovalicarb	140923-17-7	$C_{18}H_{28}N_2O_3$	17.51，17.81	134.09642	116.07060	158.11755
Carboxin	5234-68-4	$C_{12}H_{13}NO_2S$	17.59	235.06615	218.03960	143.01613
Myclobutanil	88671-89-0	$C_{15}H_{17}ClN_4$	17.59	179.02447	150.01050	245.05885
p,p'-Dichlorodiphenyldichloroethylene	72-55-9	$C_{14}H_8Cl_4$	17.63	315.93746	247.99681	245.99976
Buprofezin	69327-76-0	$C_{16}H_{23}N_3OS$	17.67	175.08659	171.09505	249.10561
Imazalil	35554-44-0	$C_{14}H_{14}Cl_2N_2O$	17.69	174.95260	172.95555	158.97628
Flusilazole	85509-19-9	$C_{16}H_{15}N_3F_2Si$	17.70	233.05926	206.05443	314.09196
Methoprotryne	841-06-5	$C_{11}H_{21}N_5OS$	17.71	256.12266	184.06514	212.09644
Azaconazole	60207-31-0	$C_{12}H_{11}N_3O_2Cl_2$	17.74	216.98176	144.96063	174.95259
Bupirimate	41483-43-6	$C_{13}H_{24}N_4O_3S$	17.78	208.14443	193.14477	273.10158
Imazamethabenz-methyl	81405-85-8	$C_{16}H_{20}N_2O_3$	17.81	144.04439	176.07060	245.09206
Kresoxim-methyl	143390-89-0	$C_{18}H_{19}NO_4$	17.82	116.04947	131.07295	206.08116
Metamitron	41394-05-2	$C_{10}H_{10}N_4O$	17.84	174.08999	173.08217	202.08491
Isoxathion	18854-01-8	$C_{13}H_{16}NO_4PS$	17.96	177.02429	159.01372	313.05322
Aramite	140-57-8	$C_{15}H_{23}O_4SCl$	17.97	185.00325	175.11174	319.07654
Nitrofen	1836-75-5	$C_{12}H_7Cl_2NO_3$	18.01	282.97975	284.97680	202.01799
Endrin	72-20-8	$C_{12}H_8CL_6O$	18.08	242.95295	280.92668	316.90336
Endrin aldehyde	7421-93-4	$C_{12}H_8OCl_6$	18.08	242.95295	280.92668	344.89827
Ancymidol	12771-68-5	$C_{15}H_{16}N_2O_2$	18.11	228.08933	107.02399	215.08150
Perthan	72-56-0	$C_{18}H_{20}Cl_2$	18.14	223.14812	178.07770	167.08552
Chlorfenapyr	122453-73-0	$C_{15}H_{11}BRCLF_3N_2O$	18.18	247.04776	363.94073	361.94277
Chloropropylate	5836-10-2	$C_{17}H_{16}O_3Cl_2$	18.37	138.99451	110.99960	251.00249
Chlorobenzilate	510-15-6	$C_{16}H_{14}O_3Cl_2$	18.37	138.99450	251.00250	252.99960
Fenthion sulfoxide	3761-41-9	$C_{10}H_{15}O_4PS_2$	18.50	294.01439	278.99091	152.98272
Diniconazole	83657-24-3	$C_{15}H_{17}N_3OCl_2$	18.54	268.00389	234.04287	165.01017
Flamprop-isopropyl	52756-22-6	$C_{19}H_{19}NO_3FCl$	18.62	276.05860	105.03349	156.00108
p,p'-Dichlorodiphenyldichloroetha	72-54-8	$C_{14}H_{10}Cl_4$	18.65	235.00758	199.03090	165.06987

ne						
Aclonifen	74070-46-5	$C_{12}H_9CLN_2O_3$	18.67	264.02962	182.06004	212.05802
o,p'-Dichlorodiphenyltrichloroethane	789-02-6	$C_{14}H_9Cl_5$	18.75	235.00758	165.06987	237.00463
Oxadixyl	77732-09-3	$C_{14}H_{18}N_2O_4$	18.77	233.09207	163.09917	132.08078
Ethion	563-12-2	$C_9H_{22}O_4P_2S_4$	18.81	230.97315	202.94185	153.01336
Mepronil	55814-41-0	$C_{17}H_{19}NO_2$	19.06	119.04914	210.06753	269.14103
Triazophos	24017-47-8	$C_{12}H_{16}N_3O_3PS$	19.21	162.06619	257.00185	172.08692
Azamethiphos	35575-96-3	$C_9H_{10}ClN_2O_5PS$	19.33	182.99558	214.96765	323.97311
Ofurace	58810-48-3	$C_{14}H_{16}NO_3Cl$	19.38	232.09681	186.09134	281.08132
Carbophenothion	786-19-6	$C_{11}H_{16}O_2PS_3Cl$	19.46	341.97331	170.96978	199.00108
Benalaxyl	71626-11-4	$C_{20}H_{23}NO_3$	19.54	148.11207	176.10699	206.11755
Tepraloxydim	149979-41-9	$C_{17}H_{24}NO_4Cl$	19.54	164.07060	136.03930	108.04439
Diofenolan	63837-33-2	$C_{18}H_{20}O_4$	19.55，19.76	186.06753	131.04914	225.09100
Cyanofenphos	13067-93-1	$C_{15}H_{14}NO_2PS$	19.59	141.00999	169.04129	185.01844
Edifenphos	17109-49-8	$C_{14}H_{15}O_2PS_2$	19.60	172.98206	186.04977	310.02455
Quinoxyfen	124495-18-7	$C_{15}H_8NOFCl_2$	19.62	306.99615	237.05844	161.00268
Endosulfan Sulfate	1031-07-8	$C_9H_6CL_6O_4S$	19.68	271.80962	236.84076	269.81257
Propiconazol	60207-90-1	$C_{15}H_{17}Cl_2N_3O_2$	19.69，19.90	172.95554	259.02871	261.02576
Norflurazon	27314-13-2	$C_{12}H_9N_3OF_3Cl$	19.73	303.03807	173.03210	302.03025
Fenhexamid	126833-17-8	$C_{14}H_{17}Cl_2NO_2$	19.78	176.97427	178.97132	301.06309
p,p'-Dichlorodiphenyltrichloroethane	50-29-3	$C_{14}H_9Cl_5$	19.83	235.00758	199.0309	165.06987
Trifloxystrobin	141517-21-7	$C_{20}H_{19}F_3N_2O_4$	19.91	116.04947	190.04986	186.05251
Hexazinone	51235-04-2	$C_{12}H_{20}N_4O_2$	20.16	171.08765	71.06037	128.08183
Tebuconazol	107534-96-3	$C_{16}H_{22}ClN_3O$	20.25	250.07417	125.01525	163.03090
Chloridazon	1698-60-8	$C_{10}H_8ClN_3O$	20.25	220.02721	221.03504	222.02426
Nuarimol	63284-71-9	$C_{17}H_{12}N_2OFCl$	20.26	235.03205	203.06152	314.06167
Diclofop-methyl	51338-27-3	$C_{16}H_{14}Cl_2O_4$	20.38	340.02637	254.97881	252.98176
Piperonyl butoxide	51-03-6	$C_{19}H_{30}O_5$	20.61	176.08318	161.0597	177.09100
Oxycarboxin	5259-88-1	$C_{12}H_{13}NO_4S$	20.67	175.00595	250.02943	267.05597
Resmethrin	10453-86-8	$C_{22}H_{26}O_3$	20.69	143.08552	128.06205	171.08044
Zoxamide	156052-68-5	$C_{14}H_{16}NO_2Cl_3$	20.82	186.97120	258.04470	242.01340
Mefenpyr-diethyl	135590-91-9	$C_{16}H_{18}Cl_2N_2O_4$	21.01	271.00355	227.01373	299.03485
Benzoylprop-ethyl	22212-55-1	$C_{18}H_{17}NO_3Cl_2$	21.11	105.03349	292.02905	260.02396
Spiromesifen	283594-90-1	$C_{23}H_{30}O_4$	21.13	254.13013	231.10033	226.13521
Endrin ketone	53494-70-5	$C_{12}H_8OCl_6$	21.15	314.90632	281.93003	242.95295
Fenamiphos sulfone	31972-44-8	$C_{13}H_{22}NO_5PS$	21.26	292.04030	320.07160	214.06244
Bromuconazole	116255-48-2	$C_{13}H_{12}BrCl_2N_3O$	21.30，22.11	172.95555	294.91096	174.95260
Fenpiclonil	74738-17-3	$C_{11}H_6N_2Cl_2$	21.31	235.99026	201.02140	237.98730
Phosmet	732-11-6	$C_{11}H_{12}NO_4PS_2$	21.36	160.03930	104.02566	133.02871
Bromopropylate	18181-80-1	$C_{17}H_{16}O_3Br_2$	21.47	184.94196	182.94400	338.90147
Tetramethrin	7696-12-0	$C_{19}H_{25}NO_4$	21.60	164.07060	107.04914	123.11682
Picolinafen	137641-05-5	$C_{19}H_{12}N_2O_2F_4$	21.60	238.04742	145.02596	376.08294

Bifenthrin	82657-04-3	$C_{23}H_{22}ClF_3O_2$	21.63	181.10118	165.06988	182.10453
Piperophos	24151-93-7	$C_{14}H_{28}NO_3PS_2$	21.67	122.09642	140.10699	320.14437
4,4'-Methoxychlor	72-43-5	$C_{16}H_{15}O_2CL_3$	21.72	227.10666	212.08318	228.11001
Bifenazate	149877-41-8	$C_{17}H_{20}N_2O_3$	21.72	300.14684	258.09989	196.07569
Fenpropathrin	39515-41-8	$C_{22}H_{23}NO_3$	21.82	181.06479	209.08350	265.07334
Etoxazole	153233-91-1	$C_{21}H_{23}F_2NO_2$	21.90	300.11945	187.11174	330.13001
Tebufenpyrad	119168-77-3	$C_{18}H_{24}N_3OCl$	21.93	333.16024	171.03197	276.08982
Fenamidone	161326-34-7	$C_{17}H_{17}N_3OS$	21.94	268.09029	206.07464	238.11006
Dicofol	115-32-2	$C_{14}H_9CL_5O$	21.97	138.99452	199.03090	140.99157
Metconazole	125116-23-6	$C_{17}H_{22}N_3OCl$	21.98	125.01525	145.06479	250.11189
Fenazaquin	120928-09-8	$C_{20}H_{22}N_2O$	22.00	145.10118	117.06988	160.12465
Tetradifon	116-29-0	$C_{12}H_6O_2SCl_4$	22.35	226.88864	228.88569	158.96659
Furathiocarb	65907-30-4	$C_{18}H_{26}N_2O_5S$	22.53	163.07535	194.03960	325.13423
Phosalone	2310-17-0	$C_{12}H_{15}NO_4PS_2Cl$	22.67	182.00033	121.04129	366.98631
Pyriproxyfen	95737-68-1	$C_{20}H_{19}NO_3$	22.87	136.07569	226.09883	137.07904
Mirex	2385-85-5	$C_{10}Cl_{12}$	22.95	271.80962	269.81257	331.80962
Mefenacet	73250-68-7	$C_{16}H_{14}N_2O_2S$	23.03	192.01137	136.02154	120.08077
Cyhalothrin	68085-85-8	$C_{23}H_{19}ClF_3NO_3$	23.12，23.48	141.05103	197.03394	161.05726
Tralkoxydim	87820-88-0	$C_{20}H_{27}NO_3$	23.16	137.04712	227.13046	283.15668
Fenarimol	60168-88-9	$C_{17}H_{12}N_2OCl_2$	23.57	251.00250	219.03197	252.99955
Trifenmorph	1420-06-0	$C_{23}H_{23}NO$	23.64	243.11682	228.09335	239.08552
Azinphos-ethyl	2642-71-9	$C_{12}H_{16}N_3O_3PS_2$	23.84	132.04439	104.04947	160.05053
Pyrazophos	13457-18-6	$C_{14}H_{20}N_3O_5PS$	23.87	221.07949	265.08794	193.04819
Acrinathrin	101007-06-1	$C_{26}H_{21}F_6NO_5$	23.89	181.06479	208.07569	289.06593
Fluoroglycofen-ethyl	77501-90-7	$C_{18}H_{13}NO_7F_3Cl$	23.95	343.99205	223.03538	447.03271
Fenoxaprop-ethyl	66441-23-4	$C_{18}H_{16}NO_5Cl$	24.24	288.04219	182.06004	361.07115
Bitertanol	55179-31-2	$C_{20}H_{23}N_3O_2$	24.58	170.07262	168.11314	171.07597
Spirodiclofen	148477-71-8	$C_{21}H_{24}Cl_2O_4$	24.73	259.05203	312.03145	156.96063
Permethrin	61949-76-6	$C_{21}H_{20}CL_2O_3$	24.77，25.02	183.08044	163.00758	127.03090
Pyridaben	96489-71-3	$C_{19}H_{25}ClN_2OS$	24.95	147.11682	117.06987	309.08228
Fluquinconazole	136426-54-5	$C_{16}H_8N_5OFCl_2$	25.12	340.03959	298.01779	286.01779
Coumaphos	56-72-4	$C_{14}H_{16}CLO_5PS$	25.15	362.01391	210.00782	225.98498
Prochloraz	67747-09-5	$C_{15}H_{16}N_3O_2Cl_3$	25.27	180.11313	265.95368	308.00063
Butafenacil	134605-64-4	$C_{20}H_{18}N_2O_6F_3Cl$	25.62	331.00918	123.99485	179.98468
Prallethrin	23031-36-9	$C_{19}H_{24}O_3$	25.82	123.11682	81.06987	105.06987
Cyfluthrin	68359-37-5	$C_{22}H_{18}NO_3FCl_2$	25.93，26.12，26.25，26.34	206.06004	199.05536	163.00758
Cypermethrin	52315-07-8	$C_{22}H_{19}Cl_2NO_3$	26.50，26.70，26.82，26.92	181.06479	163.00758	127.03090
Boscalid	188425-85-6	$C_{18}H_{12}Cl_2N_2O$	26.52	342.03212	111.99485	139.98977
Quizalofop-ethyl	76578-14-8	$C_{19}H_{17}CLN_2O_4$	26.74	372.08714	243.03196	163.00575
Flucythrinate	70124-77-5	$C_{26}H_{23}F_2NO_4$	26.92，27.29	157.04595	199.09290	225.07843
Etofenprox	80844-07-1	$C_{25}H_{28}O_3$	27.02	163.11174	135.08044	164.11510
Pyridalyl	179101-81-6	$C_{18}H_{14}NO_3F_3Cl_4$	27.17	204.06307	148.03686	176.03177

Fenvalerate	51630-58-1	$C_{25}H_{22}NO_3Cl$	28.20，28.60	419.12830	125.01525	167.06220
Flumioxazin	103361-09-7	$C_{19}H_{15}FN_2O_4$	28.24	354.10104	259.05135	326.10612
Pyraclostrobin	175013-18-0	$C_{19}H_{18}N_3O_4Cl$	28.33	132.04439	104.04947	164.07060
tau-Fluvalinate	102851-06-9	$C_{26}H_{22}ClF_3N_2O_3$	28.61，28.74	250.06049	252.05754	205.99789
Difenoconazole	119446-68-3	$C_{19}H_{17}N_3O_3Cl_2$	28.94，29.06	323.02363	266.97881	264.98176
Deltamethrin	52918-63-5	$C_{22}H_{19}Br_2NO_3$	29.20，29.55	171.98821	173.98617	252.90450
Azoxystrobin	131860-33-8	$C_{22}H_{17}N_3O_5$	30.01	344.10296	372.09788	388.09279
Dimethomorph	110488-70-5	$C_{21}H_{22}ClNO_4$	30.05	301.06260	303.05965	387.12319

Table S2. Matrixeffect (ME) obtained for the target compounds in the orange juice and celery juice matrix.

Pesticides	orange juice			celery juice		
	**ME[a]**	**ME[b]**	**ME[c]**	**ME[a]**	**ME[b]**	**ME[c]**
Clopyralid	1.53	1.53	1.51	1.35	1.21	1.13
Dichlorvos	1.26	1.02	1.04	1.10	1.74	1.09
Methamidophos	1.55	1.02	1.90	1.18	1.51	1.15
Thiofanox	1.13	1.19	1.96	1.25	1.55	1.37
Allidochlor	1.12	1.15	1.76	1.38	1.33	1.01
Dichlobenil	1.01	1.01	1.73	1.33	1.24	1.79
EPTC	1.20	1.03	1.61	1.07	1.25	1.59
Dichlormid	1.06	1.29	1.03	1.14	1.03	1.54
2,4,6-Trichlorophenol	1.51	1.38	1.87	1.48	1.36	1.84
3,5-Dichloroaniline	1.05	1.08	1.23	1.07	1.16	1.77
O-Phthalimide	1.33	1.29	1.86	1.11	1.39	1.02
Mevinphos	1.60	1.59	1.62	1.59	1.51	1.53
Acephate	1.04	1.41	1.36	1.43	1.71	1.39
Vernolate	1.41	1.32	1.34	1.44	1.50	1.54
Propham	1.24	1.42	1.91	1.39	1.17	1.18
Etridiazole	1.10	1.28	1.35	1.27	1.35	1.20
Pebulate	1.32	1.56	1.84	1.09	1.08	1.30
cis-1,2,3,6-Tetrahydrophthalimide	1.23	1.65	1.09	1.14	1.72	1.92
Chloroneb	1.25	1.48	1.87	1.42	1.19	1.27
Tebuthiuron	1.14	1.75	1.41	1.54	1.11	1.89
Fenobucarb	1.03	1.01	1.55	1.33	1.71	1.83

Pentachlorobenzene	1.10	1.18	1.64	1.45	1.50	1.35
Isoprocarb	1.57	1.63	1.59	1.15	1.43	1.43
Molinate	1.44	1.44	1.16	1.48	1.65	1.78
Heptenophos	1.40	1.33	1.78	1.17	1.51	1.75
Chlorfenprop-methyl	1.49	1.08	1.26	1.31	1.63	1.14
Omethoate	1.33	1.72	1.05	1.21	1.23	1.36
Propoxur	1.13	1.34	1.55	1.59	1.15	1.58
Tecnazene	1.44	1.34	1.07	1.06	1.66	1.05
Propachlor	1.50	1.11	1.12	1.43	1.76	1.78
Diphenylamine	1.03	1.11	1.93	1.32	1.05	1.29
Ethoprophos	1.39	1.72	1.70	1.38	1.20	1.55
Cycloate	1.47	1.28	1.47	1.01	1.32	1.81
2,3,5,6-tetrachloroaniline	1.21	1.08	1.32	1.46	1.26	1.01
Atrazine-desethyl	1.18	1.36	1.77	1.08	1.16	1.63
Dicrotophos	1.36	1.58	1.41	1.19	1.02	1.21
Methabenzthiazuron	1.13	1.57	1.21	1.34	1.12	1.58
Trifluralin	1.03	1.62	1.16	1.56	1.64	1.98
Bendiocarb	1.44	1.30	1.42	1.58	1.51	1.84
Benfluralin	1.56	1.39	1.48	1.35	1.21	1.37
Sulfotep	1.00	1.07	1.65	1.50	1.32	1.30
Cadusafos	1.10	1.17	1.02	1.11	1.43	1.11
Tebutam	1.02	1.05	2.00	1.33	1.34	1.14
Promecarb	1.10	1.73	1.90	1.28	1.66	1.26
Phorate	1.56	1.43	1.58	1.41	1.27	1.31
Atratone	1.07	1.45	1.40	1.40	1.36	1.12

3,4,5-Trimethacarb	1.06	1.14	1.01	1.10	1.34	1.76
Dicloran	1.14	1.23	1.57	1.37	1.06	1.47
Pentachloroanisole	1.46	1.51	1.93	1.03	1.40	1.19
Ethoxyquin	1.52	1.71	1.16	1.17	1.33	1.42
Prometon	1.14	1.58	1.96	1.45	1.25	1.57
Atrazine	1.12	1.35	1.61	1.24	1.31	1.37
Monolinuron	1.40	1.17	1.05	1.14	1.65	1.86
Propazine	1.14	1.29	1.56	1.41	1.52	1.57
Clomazone	1.11	1.35	1.76	1.33	1.09	1.78
α-Hexachlorocyclohexane	1.35	1.34	1.03	1.47	1.14	1.72
Terbumeton	1.29	1.59	1.43	1.52	1.62	1.61
β-Hexachlorocyclohexane	1.55	1.57	1.23	1.06	1.23	1.64
Aminocarb	1.22	1.41	1.34	1.41	1.07	1.42
Isocarbamid	1.25	1.22	1.80	1.58	1.46	1.28
Cyromazine	1.52	1.73	1.93	1.43	1.36	1.60
γ-Hexachlorocyclohexane	1.47	1.73	1.90	1.16	1.27	1.48
Propetamphos	1.43	1.16	1.75	1.12	1.51	1.24
Cycluron	1.11	1.62	1.06	1.28	1.67	1.19
Terbuthylazine	1.51	1.24	1.07	1.53	1.71	1.59
Terbufos	1.13	1.08	1.16	1.42	1.29	1.86
Cyanophos	1.52	1.10	1.13	1.22	1.74	1.53
Trietazine	1.49	1.66	1.99	1.27	1.03	1.13
Quintozene	1.51	1.25	1.98	1.05	1.01	1.04
Fonofos	1.52	1.12	1.99	1.26	1.40	1.76
Pyroquilon	1.43	1.79	1.91	1.04	1.57	1.51

Dinoterb	1.06	1.54	1.41	1.19	1.66	1.10
Pyrimethanil	1.09	1.49	1.46	1.17	1.50	1.05
Diazinon	1.25	1.73	1.84	1.04	1.57	1.87
Flufenoxuron	1.19	1.28	1.46	1.45	1.48	1.09
Disulfoton	1.16	1.66	1.09	1.29	1.58	1.63
Paraoxon-methyl	1.05	1.78	1.69	1.01	1.00	1.51
Secbumeton	1.28	1.78	1.17	1.05	1.57	1.52
Aziprotryne	1.55	1.48	1.47	1.02	1.55	1.62
Dinitramine	1.32	1.11	1.39	1.12	1.69	1.05
Fenfuram	1.37	1.15	1.60	1.34	1.44	1.64
δ-Hexachlorocyclohexane	1.47	1.44	1.98	1.57	1.09	1.48
Mexacarbate	1.34	1.74	1.71	1.44	1.61	1.14
Isazofos	1.22	1.05	1.06	1.16	1.04	1.45
Chlorothalonil	1.26	1.76	1.91	1.50	1.49	1.84
Triallate	1.29	1.27	1.70	1.05	1.37	1.94
Tebupirimfos	1.42	1.22	1.42	1.27	1.73	1.48
musk ambrette	1.03	1.49	1.58	1.54	1.00	1.52
Oxabetrinil	1.24	1.49	1.53	1.58	1.14	1.43
Iprobenfos	1.44	1.17	1.88	1.23	1.54	1.16
Fluroxypyr	1.56	1.69	1.48	1.25	1.22	1.97
Pirimicarb	1.51	1.47	1.02	1.02	1.08	1.76
Monalide	1.41	1.04	1.87	1.30	1.68	1.43
Furmecyclox	1.56	1.23	1.97	1.32	1.16	1.94
Benoxacor	1.16	1.16	1.73	1.18	1.39	1.60
Pentachloroaniline	1.31	1.35	1.90	1.30	1.54	1.40

Benfuresate	1.33	1.57	1.16	1.22	1.64	1.40
Dioxacarb	1.42	1.65	1.54	1.55	1.39	1.35
Cyprazine	1.04	1.01	1.69	1.47	1.10	1.77
Phosphamidon	1.29	1.36	1.77	1.49	1.67	1.74
Dichlorprop	1.31	1.25	1.77	1.35	1.53	1.85
Dichlofenthion	1.45	1.47	1.77	1.54	1.46	1.56
Fenthion	1.60	1.59	1.33	1.48	1.10	1.08
Propanil	1.19	1.49	1.15	1.38	1.44	1.96
2,4-DB	1.58	1.63	1.03	1.42	1.06	1.02
Chlorthiamid	1.12	1.14	1.11	1.10	1.53	1.90
Dimethachlor	1.36	1.25	1.57	1.02	1.48	1.33
Metribuzin	1.37	1.78	1.65	1.43	1.65	1.25
Dimethenamid	1.44	1.20	1.45	1.12	1.06	1.68
Bromobutide	1.51	1.63	1.74	1.57	1.01	1.20
Terbucarb	1.42	1.60	1.70	1.29	1.04	1.53
Malaoxon	1.45	1.21	1.30	1.26	1.13	1.28
Vinclozolin	1.06	1.23	1.76	1.25	1.74	1.14
Parathion-methyl	1.44	1.04	1.49	1.09	1.32	1.46
Chlorpyrifos-methyl	1.46	1.25	1.36	1.31	1.39	1.38
Transfluthrin	1.52	1.38	1.36	1.08	1.13	1.71
Simetryn	1.21	1.18	1.18	1.11	1.50	1.63
Fuberidazole	1.44	1.15	1.06	1.52	1.23	1.68
Tolclofos-methyl	1.19	1.78	1.33	1.37	1.23	1.69
Alachlor	1.49	1.60	1.14	1.35	1.74	1.59
Ametryn	1.39	1.47	1.32	1.01	1.57	1.19

Heptachlor	1.09	1.16	1.14	1.54	1.78	1.36
Prometryn	1.08	1.30	1.71	1.16	1.37	1.66
Acetochlor	1.19	1.06	1.22	1.39	1.61	1.27
Paraoxon-ethyl	1.13	1.57	1.52	1.43	1.64	1.93
Metalaxyl	1.24	1.53	1.24	1.11	1.59	1.24
Tridiphane	1.53	1.14	1.22	1.43	1.73	1.16
Octachlorodipropyl ether	1.51	1.77	1.34	1.06	1.33	1.33
Prosulfocarb	1.40	1.73	1.42	1.05	1.21	1.96
Fenpropidin	1.32	1.53	1.60	1.52	1.38	1.30
1-Naphthylacetamide	1.03	1.67	1.17	1.48	1.68	1.37
Dithiopyr	1.30	1.57	1.58	1.56	1.61	1.06
Orbencarb	1.56	1.27	1.24	1.52	1.02	1.69
Terbutryn	1.21	1.50	1.53	1.49	1.37	1.72
Spiroxamine	1.34	1.10	1.51	1.10	1.44	1.71
Methiocarb	1.36	1.34	1.63	1.53	1.17	1.12
Fenitrothion	1.24	1.35	1.30	1.20	1.71	1.84
Pirimiphos-methyl	1.09	1.13	1.20	1.20	1.40	1.62
Methiocarb sulfone	1.05	1.55	1.43	1.09	1.74	1.13
Ethofumesate	1.57	1.30	1.51	1.60	1.37	1.61
Linuron	1.38	1.45	1.06	1.56	1.29	1.65
Probenazole	1.03	1.49	1.64	1.45	1.50	1.21
Noruron	1.22	1.77	1.79	1.03	1.55	1.74
Quinoclamine	1.02	1.44	1.73	1.25	1.67	1.99
Dipropetryn	1.27	1.16	1.42	1.30	1.69	1.45
Malathion	1.19	1.40	1.18	1.48	1.80	1.67

Thiobencarb	1.50	1.21	1.96	1.56	1.32	1.57
Diethofencarb	1.40	1.19	1.77	1.51	1.77	1.20
Phorate sulfoxide	1.21	1.13	1.39	1.57	1.68	1.44
Metolachlor	1.26	1.47	1.64	1.34	1.75	1.21
Fenpropimorph	1.21	1.36	1.27	1.52	1.26	1.97
Cyanazine	1.11	1.24	1.70	1.22	1.54	1.40
Chlorpyrifos	1.10	1.15	1.63	1.12	1.26	1.37
Parathion	1.39	1.58	1.19	1.16	1.54	1.78
Flufenacet	1.23	1.20	1.67	1.05	1.68	1.25
Rabenzazol	1.13	1.67	1.40	1.45	1.23	1.46
4,4'-Dichlorobenzophenone	1.23	1.19	1.84	1.31	1.46	1.76
Triadimefon	1.59	1.49	1.40	1.08	1.38	1.17
Chlorthal-dimethyl	1.25	1.03	1.38	1.20	1.00	1.59
Dicapthon	1.52	1.15	1.07	1.46	1.57	1.74
Isofenphos-oxon	1.15	1.48	1.46	1.03	1.42	1.91
Isocarbophos	1.10	1.06	1.45	1.51	1.78	1.33
Tetraconazole	1.03	1.12	1.46	1.05	1.40	1.60
Isobenzan	1.19	1.50	1.28	1.27	1.34	1.13
Flurochloridone	1.40	1.13	1.40	1.50	1.10	1.64
Fenson	1.16	1.61	1.28	1.49	1.29	1.57
Pyracarbolid	1.29	1.20	1.75	1.05	1.74	1.43
Dodemorph	1.35	1.20	1.14	1.20	1.20	1.41
Mgk 264	1.42	1.42	1.73	1.59	1.31	1.31
Butralin	1.55	1.35	1.74	1.09	1.75	1.23
Carbaryl	1.13	1.47	1.21	1.04	1.19	1.75

Diphenamid	1.48	1.29	1.59	1.53	1.61	1.47
Pirimiphos-ethyl	1.13	1.14	1.99	1.43	1.31	1.07
Isodrin	1.40	1.51	1.49	1.09	1.56	1.66
Aldrin	1.25	1.03	1.04	1.45	1.21	1.05
Isopropalin	1.58	1.09	1.71	1.18	1.14	1.07
Cyprodinil	1.06	1.09	1.98	1.43	1.06	1.63
Isofenphos-methyl	1.55	1.01	1.52	1.23	1.19	1.76
Octachlorostyrene	1.57	1.77	1.38	1.55	1.01	1.14
Metazachlor	1.13	1.01	1.08	1.14	1.01	1.90
Dimethametryn	1.01	1.21	1.40	1.43	1.27	1.73
Pendimethalin	1.07	1.46	1.97	1.56	1.60	1.74
Disulfoton-sulfone	1.45	1.64	1.61	1.15	1.09	1.31
Phorate sulfone	1.16	1.34	1.32	1.52	1.12	1.72
Terbufos sulfone	1.42	1.01	1.19	1.50	1.72	1.58
Paclobutrazol	1.15	1.69	1.44	1.34	1.17	1.47
Penconazole	1.01	1.80	1.08	1.30	1.27	1.72
Chlozolinate	1.01	1.25	1.62	1.59	1.29	1.45
Pyrifenox	1.17	1.14	1.46	1.07	1.21	1.15
Tolylfluanid	1.54	1.44	1.25	1.51	1.21	1.04
Fosthiazate	1.29	1.28	1.78	1.39	1.44	1.75
Phosfolan	1.10	1.34	1.54	1.39	1.01	1.27
Allethrin	1.45	1.30	1.51	1.23	1.55	1.01
Isofenphos	1.53	1.14	1.97	1.17	1.02	1.47
Captan	1.03	1.53	1.03	1.19	1.41	1.01
Fipronil	1.05	1.47	1.76	1.24	1.00	1.33

Diclocymet	1.22	1.17	1.51	1.51	1.51	1.34
Quinalphos	1.23	1.58	1.63	1.37	1.36	1.05
Phenthoate	1.49	1.58	1.11	1.07	1.04	1.88
Triadimenol	1.50	1.17	1.81	1.53	1.36	1.12
Dinobuton	1.45	1.62	1.37	1.11	1.32	1.29
Furalaxyl	1.41	1.65	1.00	1.32	1.11	1.32
Crotoxyphos	1.16	1.16	1.98	1.30	1.39	1.37
Procymidone	1.11	1.05	1.08	1.40	1.18	1.10
Chlorbenside	1.07	1.70	1.74	1.37	1.65	1.23
Chlorflurenol-methyl	1.47	1.43	1.52	1.26	1.27	1.57
Chlordane	1.49	1.02	1.19	1.21	1.62	1.37
Methidathion	1.45	1.42	1.54	1.14	1.10	1.52
Haloxyfop-methyl	1.07	1.50	1.36	1.52	1.35	1.23
Bromophos-ethyl	1.07	1.71	1.33	1.03	1.69	1.37
Procyazine	1.14	1.14	1.15	1.53	1.78	1.48
Disulfoton-sulfoxide	1.18	1.01	1.41	1.18	1.08	1.99
Tetrachlorvinphos	1.48	1.58	1.40	1.27	1.27	1.37
Endosulfan	1.20	1.29	1.36	1.07	1.42	1.13
Mepanipyrim	1.16	1.76	1.26	1.24	1.05	1.92
Butachlor	1.01	1.61	1.29	1.47	1.02	1.25
Ditalimfos	1.46	1.41	1.05	1.38	1.25	1.75
TCMTB	1.12	1.15	1.36	1.07	1.03	1.44
trans-Nonachlor	1.32	1.48	1.19	1.36	1.07	1.01
Chlorfenson	1.40	1.67	1.07	1.16	1.64	1.81
Fenamiphos	1.22	1.60	1.20	1.17	1.66	1.27

Picoxystrobin	1.20	1.68	1.47	1.37	1.71	1.41
Napropamide	1.19	1.50	1.26	1.35	1.17	1.57
Hexaconazole	1.14	1.80	1.16	1.18	1.57	1.84
Flutolanil	1.05	1.51	1.03	1.43	1.79	1.38
Prothiophos	1.44	1.28	1.01	1.01	1.52	1.55
Isoprothiolane	1.44	1.19	1.06	1.17	1.76	1.82
Profenofos	1.54	1.18	1.69	1.58	1.43	1.46
tricyclazole	1.43	1.73	1.35	1.59	1.12	1.94
Pretilachlor	1.13	1.35	1.25	1.20	1.69	1.22
Dieldrin	1.04	1.79	1.61	1.41	1.41	1.07
Oxadiazon	1.59	1.59	1.72	1.49	1.69	1.47
Iprovalicarb	1.03	1.01	1.08	1.26	1.73	1.28
Carboxin	1.46	1.36	1.50	1.21	1.33	1.46
Myclobutanil	1.53	1.56	1.55	1.38	1.38	1.98
p,p'-Dichlorodiphenyldichloroethylene	1.16	1.22	1.68	1.41	1.50	1.64
Buprofezin	1.13	1.14	1.39	1.33	1.70	1.32
Imazalil	1.50	1.78	1.69	1.25	1.46	1.61
Flusilazole	1.34	1.49	1.20	1.16	1.15	1.23
Methoprotryne	1.30	1.34	1.12	1.17	1.54	1.12
Azaconazole	1.43	1.36	1.61	1.48	1.60	1.17
Bupirimate	1.40	1.15	1.92	1.43	1.48	1.48
Imazamethabenz-methyl	1.54	1.66	1.89	1.58	1.61	1.96
Kresoxim-methyl	1.46	1.10	1.01	1.02	1.55	1.51
Metamitron	1.00	1.02	1.06	1.24	1.25	1.22
Isoxathion	1.57	1.53	1.29	1.40	1.39	1.60

Aramite	1.21	1.09	1.43	1.54	1.63	1.96
Nitrofen	1.04	1.67	1.35	1.11	1.79	1.38
Endrin	1.37	1.36	1.91	1.47	1.02	1.23
Endrin aldehyde	1.32	1.78	1.26	1.12	1.01	1.83
Ancymidol	1.05	1.27	1.70	1.57	1.08	1.07
Perthan	1.42	1.47	1.36	1.03	1.05	1.64
Chlorfenapyr	1.02	1.74	1.53	1.04	1.59	1.63
Chloropropylate	1.22	1.52	1.12	1.19	1.54	1.37
Chlorobenzilate	1.41	1.08	1.11	1.16	1.21	1.53
Fenthion sulfoxide	1.39	1.11	1.42	1.49	1.16	1.46
Diniconazole	1.53	1.67	1.15	1.07	1.43	1.31
Flamprop-isopropyl	1.39	1.27	1.84	1.39	1.64	1.23
p,p'-Dichlorodiphenyldichloroethane	1.54	1.26	1.89	1.56	1.15	1.08
Aclonifen	1.03	1.33	1.22	1.45	1.06	1.41
o,p'-Dichlorodiphenyltrichloroethane	1.34	1.42	1.78	1.14	1.73	1.79
Oxadixyl	1.26	1.30	1.85	1.22	1.52	1.82
Ethion	1.40	1.73	1.97	1.16	1.04	1.46
Mepronil	1.39	1.02	1.85	1.17	1.53	1.33
Triazophos	1.17	1.48	1.03	1.22	1.06	1.47
Azamethiphos	1.12	1.70	1.31	1.48	1.56	1.64
Ofurace	1.56	1.41	1.46	1.01	1.05	1.34
Carbophenothion	1.21	1.29	1.08	1.30	1.06	1.49
Benalaxyl	1.49	1.25	1.54	1.00	1.16	1.39
Tepraloxydim	1.38	1.03	1.92	1.27	1.79	1.13
Diofenolan	1.00	1.01	1.55	1.08	1.54	1.91

Cyanofenphos	1.13	1.75	1.51	1.20	1.56	1.73
Edifenphos	1.50	1.22	1.65	1.57	1.71	1.62
Quinoxyfen	1.02	1.12	1.08	1.54	1.74	1.99
Endosulfan Sulfate	1.29	1.11	1.99	1.50	1.10	1.87
Propiconazol	1.39	1.50	1.62	1.48	1.17	1.68
Norflurazon	1.27	1.05	1.88	1.43	1.50	1.02
Fenhexamid	1.35	1.36	1.26	1.55	1.76	1.49
p,p'-Dichlorodiphenyltrichloroethane	1.15	1.79	1.28	1.50	1.29	1.25
Trifloxystrobin	1.02	1.05	1.94	1.25	1.30	1.26
Hexazinone	1.54	1.66	1.00	1.00	1.21	1.54
Tebuconazol	1.18	1.04	1.49	1.55	1.05	1.01
Chloridazon	1.05	1.80	1.25	1.22	1.14	1.86
Nuarimol	1.28	1.78	1.36	1.31	1.24	1.70
Diclofop-methyl	1.55	1.69	1.99	1.05	1.59	1.08
Piperonyl butoxide	1.09	1.49	1.53	1.11	1.22	1.16
Oxycarboxin	1.03	1.44	1.88	1.23	1.24	1.88
Resmethrin	1.50	1.41	1.59	1.40	1.18	1.18
Zoxamide	1.13	1.65	1.35	1.48	1.49	1.90
Mefenpyr-diethyl	1.11	1.74	1.84	1.44	1.30	1.32
Benzoylprop-ethyl	1.39	1.41	1.91	1.13	1.37	1.80
Spiromesifen	1.45	1.14	1.48	1.20	1.18	1.06
Endrin ketone	1.14	1.65	1.64	1.10	1.09	1.43
Fenamiphos sulfone	1.58	1.76	1.11	1.38	1.47	1.28
Bromuconazole	1.56	1.18	1.88	1.32	1.64	1.45
Fenpiclonil	1.00	1.04	1.20	1.57	1.53	1.79

Phosmet	1.20	1.55	1.33	1.22	1.09	1.69
Bromopropylate	1.00	1.24	1.84	1.38	1.51	1.16
Tetramethrin	1.46	1.63	1.37	1.43	1.58	1.64
Picolinafen	1.02	1.20	1.06	1.21	1.43	1.23
Bifenthrin	1.27	1.62	1.56	1.45	1.33	1.79
Piperophos	1.23	1.70	1.25	1.41	1.07	1.99
4,4'-Methoxychlor	1.01	1.50	1.57	1.52	1.79	1.83
Bifenazate	1.07	1.33	1.34	1.02	1.24	1.17
Fenpropathrin	1.50	1.65	1.77	1.48	1.44	1.68
Etoxazole	1.04	1.48	1.92	1.31	1.44	1.00
Tebufenpyrad	1.09	1.05	1.66	1.13	1.29	1.11
Fenamidone	1.56	1.19	1.19	1.21	1.70	1.64
Dicofol	1.48	1.58	1.31	1.20	1.23	1.87
Metconazole	1.47	1.33	1.07	1.23	1.27	1.59
Fenazaquin	1.09	1.04	1.67	1.11	1.17	1.65
Tetradifon	1.58	1.80	1.03	1.59	1.72	1.02
Furathiocarb	1.10	1.62	1.18	1.17	1.18	1.83
Phosalone	1.17	1.28	1.26	1.56	1.67	1.30
Pyriproxyfen	1.43	1.09	1.51	1.27	1.05	1.18
Mirex	1.50	1.57	1.67	1.00	1.55	1.68
Mefenacet	1.01	1.58	1.95	1.11	1.36	1.40
Cyhalothrin	1.03	1.55	1.26	1.26	1.26	1.25
Tralkoxydim	1.56	1.75	1.24	1.01	1.41	1.16
Fenarimol	1.35	1.72	1.55	1.25	1.58	1.98
Trifenmorph	1.17	1.62	1.66	1.28	1.76	1.87

Azinphos-ethyl	1.57	1.31	1.15	1.09	1.18	1.55
Pyrazophos	1.59	1.34	1.46	1.40	1.57	1.29
Acrinathrin	1.18	1.70	1.51	1.51	1.46	1.11
Fluoroglycofen-ethyl	1.38	1.27	1.98	1.31	1.70	1.09
Fenoxaprop-ethyl	1.09	1.52	1.69	1.31	1.68	1.98
Bitertanol	1.19	1.23	1.48	1.27	1.30	1.25
Spirodiclofen	1.35	1.68	1.05	1.31	1.37	1.93
Permethrin	1.30	1.77	1.77	1.06	1.70	1.61
Pyridaben	1.07	1.07	1.33	1.05	1.45	1.26
Fluquinconazole	1.11	1.46	1.03	1.40	1.66	1.61
Coumaphos	1.42	1.56	1.08	1.15	1.73	1.95
Prochloraz	1.30	1.73	1.42	1.31	1.58	1.25
Butafenacil	1.52	1.06	1.44	1.09	1.74	1.01
Prallethrin	1.32	1.14	1.90	1.15	1.80	1.63
Cyfluthrin	1.24	1.25	1.79	1.29	1.19	1.58
Cypermethrin	1.38	1.43	1.02	1.14	1.64	1.87
Boscalid	1.00	1.53	1.61	1.45	1.19	1.81
Quizalofop-ethyl	1.31	1.65	1.71	1.28	1.11	1.96
Flucythrinate	1.13	1.52	1.55	1.03	1.29	1.44
Etofenprox	1.32	1.32	1.82	1.22	1.65	1.95
Pyridalyl	1.55	1.50	1.55	1.33	1.61	1.92
Fenvalerate	1.03	1.78	1.34	1.46	1.33	2.00
Flumioxazin	1.48	1.43	1.23	1.36	1.40	1.29
Pyraclostrobin	1.06	1.48	1.74	1.38	1.56	1.17
tau-Fluvalinate	1.01	1.40	1.96	1.51	1.29	1.13

Difenoconazole	1.40	1.64	1.59	1.52	1.76	1.92
Deltamethrin	1.42	1.39	1.55	1.49	1.01	1.48
Azoxystrobin	1.47	1.65	1.15	1.32	1.21	1.34
Dimethomorph	1.53	1.60	1.96	1.19	1.48	1.82

[a]ME: matrix effects are expressed as the ratio between the calibration curve slopes of maix-matched standards (m-PFC cleanup) and solvent-based standards.

[b] ME: matrix effects are expressed as the ratio between the calibration curve slopes of maix-matched standards (QuEChERS cleanup/complex matrix) and solvent-based standards.

[c] ME: matrix effects are expressed as the ratio between the calibration curve slopes of maix-matched standards (QuEChERS cleanup/simple matrix) and solvent-based standards.

Table S3. Correlation coefficients (R^2), limits of detection, spiked recoveries and RSDs of the 350 pesticides in orange juice and celery juice (n=6).

Pesticides	orange juice						celery juice					
	Correlation coefficient R^2	LOD/ (μg/kg)	LOQ/ (μg/kg)	Recoveries (%) 10 μg/kg	100 μg/kg	500 μg/kg	Correlation coefficient R^2	LOD/ (μg/kg)	LOQ/ (μg/kg)	Recoveries (%) 10 μg/kg	100 μg/kg	500 μg/kg
Clopyralid	0.9933	3.0	10.0	78.4(9.1)	78.0(6.2)	82.3(5.4)	0.9923	3.0	10.0	74.5(5.2)	81.4(7.2)	84.1(7.1)
Dichlorvos	0.9924	3.0	10.0	73.4(7.1)	74.2(9.3)	77.1(6.5)	0.9946	3.0	10.0	75.8(6.2)	79.4(10.4)	79.1(9.5)
Methamidophos	0.9990	1.0	3.0	73.2(9.2)	78.3(8.2)	84.3(5.3)	0.9914	3.0	10.0	74.3(9.1)	76.4(7.2)	78.7(4.3)
Thiofanox	0.9969	1.0	3.0	78.2(7.2)	83.1(9.3)	96.2(7.1)	0.9982	1.0	3.0	102.2(8.1)	81.8(6.8)	88.4(6.1)
Allidochlor	0.9987	0.3	1.0	91.2(6.4)	92.1(4.2)	85.3(7.4)	0.9911	0.3	1.0	103.4(6.2)	82.2(5.2)	95.5(4.0)
Dichlobenil	0.9927	0.3	1.0	103.8(7.1)	104.1(4.0)	94.4(5.6)	0.9950	0.3	1.0	79.8(9.4)	88.4(5.1)	92.2(6.3)
EPTC	0.9928	0.3	1.0	95.4(6.1)	95.3(4.3)	94.5(2.4)	0.9941	0.3	1.0	105.1(5.1)	87.8(6.4)	88.5(7.4)
Dichlormid	0.9935	0.3	1.0	102.4(6.2)	87.4(5.3)	102.5(5.6)	0.9965	0.3	1.0	92.2(6.6)	93.1(6.7)	102.4(6.2)
2,4,6-Trichlorophenol	0.9914	0.5	2.0	94.3(9.2)	84.3(5.4)	81.5(7.7)	0.9954	0.5	2.0	87.2(7.2)	99.1(8.3)	96.3(6.2)
3,5-Dichloroaniline	0.9985	0.5	2.0	107.2(8.1)	106.2(6.5)	95.2(3.9)	0.9993	0.5	2.0	95.4(5.1)	87.6(5.1)	88.4(9.5)
O-Phthalimide	0.9943	0.3	1.0	104.3(5.1)	89.4(4.2)	91.7(5.3)	0.9953	0.3	1.0	103.1(6.1)	101.5(4.6)	93.1(8.2)
Mevinphos	0.9979	1.0	3.0	74.2(6.4)	87.2(7.4)	101.4(4.3)	0.9902	3.0	10.0	72.4(4.2)	93.4(4.1)	103.3(8.1)
Acephate	0.9922	0.3	1.0	101.4(6.1)	109.4(9.1)	82.55(6.1)	0.9980	0.3	1.0	79.5(9.4)	80.5(7.7)	86.3(5.5)
Vernolate	0.9995	0.3	1.0	105.3(8.1)	76.5(8.1)	81.0(7.3)	0.9934	0.3	1.0	102.1(9.3)	95.5(6.1)	93.2(9.2)
Propham	0.9931	0.3	1.0	94.5(7.2)	110.2(8.1)	107.2(5.8)	0.9982	0.5	2.0	95.1(6.3)	98.4(7.5)	97.2(5.2)
Etridiazole	0.9951	1.0	3.0	107.1(7.5)	87.3(7.4)	97.2(6.2)	0.9929	1.0	3.0	91.4(4.5)	92.3(6.3)	105.1(7.1)
Pebulate	0.9937	1.0	3.0	101.3(5.2)	79.2(9.1)	96.5(7.8)	0.9992	1.0	3.0	106.6(8.3)	104.1(7.6)	102.1(5.2)
cis-1,2,3,6-Tetrahydrophthalimide	0.9958	0.5	2.0	96.5(5.5)	83.4(9.2)	80.2(9.1)	0.9987	0.5	2.0	94.1(8.3)	95.3(6.5)	93.4(9.3)

Chloroneb	0.9943	1.0	3.0	104.3(7.4)	80.5(9.6)	95.4(5.2)	0.9975	1.0	3.0	108.6(5.7)	102.2(5.1)	109.2(7.3)
Tebuthiuron	0.9979	0.3	1.0	107.4(7.4)	85.5(9.3)	96.4(5.5)	0.9933	0.3	1.0	79.4(10.1)	103.1(7.3)	104.1(7.2)
Fenobucarb	0.9989	1.0	3.0	95.1(6.3)	93.2(9.1)	106.3(9.1)	0.9950	1.0	3.0	83.4(9.1)	79.4(9.3)	94.5(6.4)
Pentachlorobenzene	0.9968	0.5	2.0	89.1(8.2)	74.3(9.4)	96.2(8.3)	0.9952	0.5	2.0	82.8(8.6)	1054(9.1)	102.5(9.6)
Isoprocarb	0.9952	0.5	2.0	92.4(2.2)	75.6(9.7)	107.5(6.6)	0.9947	0.5	2.0	84.1(7.2)	94.3(4.4)	84.8(6.9)
Molinate	0.9952	0.5	2.0	85.0(7.1)	107.2(9.3)	117.2(7.1)	0.9962	0.5	2.0	93.7(9.2)	118.1(8.4)	123.4(9.5)
Heptenophos	0.9920	0.5	2.0	95.4(6.1)	80.2(9.4)	92.1(9.2)	0.9937	0.5	2.0	111.6(7.7)	103.8(8.9)	105.1(7.4)
Chlorfenprop-methyl	0.9965	1.0	3.0	94.3(9.4)	73.5(9.6)	107.4(8.5)	0.9969	10	3.0	97.2(9.1)	94.2(9.3)	84.7(8.2)
Omethoate	0.9993	1.0	3.0	84.6(7.3)	75.8(9.2)	106.6(7.7)	0.9915	3.0	10.0	101.3(5.3)	84.4(9.5)	94.0(8.1)
Propoxur	0.9951	0.3	1.0	90.5(8.4)	99.5(9.1)	96.9(7.2)	0.9980	0.3	1.0	85.5(4.6)	90.7(8.8)	105.2(4.3)
Tecnazene	0.9946	0.5	2.0	105.3(7.1)	81.2(9.5)	98.6(7.7)	0.9981	0.5	2.0	97.5(9.2)	103.4(8.5)	106.5(7.4)
Propachlor	0.9951	1.0	3.0	92.5(6.6)	90.4(4.2)	94.5(5.2)	0.9908	3.0	10.0	81.6(9.5)	92.3(6.1)	94.5(5.3)
Diphenylamine	0.9962	0.3	1.0	93.4(6.1)	94.2(4.5)	100.2(6.3)	0.9982	0.3	1.0	90.4(4.3)	97.2(5.3)	96.9(6.2)
Ethoprophos	0.9942	3.0	10.0	103.4(4.5)	75.6(9.7)	107.9(8.1)	0.9968	3.0	10.0	94.5(6.6)	83.7(8.8)	84.2(8.3)
Cycloate	0.9973	0.5	2.0	109.5(7.6)	92.7(4.8)	96.9(8.0)	0.9944	0.5	2.0	87.5(4.6)	104.7(5.8)	93.3(8.2)
2,3,5,6-tetrachloroaniline	0.9929	1.0	3.0	97.4(9.5)	84.6(9.7)	93.1(7.3)	0.9992	1.0	3.0	83.5(4.2)	103.3(7.3)	108.8(9.3)
Atrazine-desethyl	0.9949	0.3	1.0	101.1(4.3)	94.4(6.3)	103.1(4.2)	0.9923	0.5	2.0	102.3(9.6)	94.4(7.2)	95.5(4.2)
Dicrotophos	0.9950	1.0	3.0	85.3(4.4)	84.5(8.1)	105.5(7.2)	0.9941	1.0	3.0	96.4(7.1)	92.5(8.3)	107.3(5.2)
Methabenzthiazuron	0.9916	0.5	2.0	94.2(8.2)	97.1(4.4)	113.4(7.2)	0.9992	0.5	2.0	98.2(11.3)	95.8(6.6)	108.6(8.3)
Trifluralin	0.9927	0.3	1.0	104.5(4.4)	83.5(2.4)	97.3(9.1)	0.9936	0.3	1.0	78.5(7.2)	104.1(4.4)	109.4(9.5)
Bendiocarb	0.9999	0.5	2.0	92.3(8.2)	102.4(9.4)	101.3(4.4)	0.9900	0.5	2.0	97.5(9.2)	92.5(8.4)	95.3(6.4)
Benfluralin	0.9974	0.3	1.0	107.5(9.4)	96.5(7.1)	88.2(4.5)	0.9978	0.3	1.0	74.4(8.1)	103.3(8.5)	88.3(4.2)
Sulfotep	0.9970	0.5	2.0	92.3(7.4)	98.4(7.2)	94.2(8.2)	0.9956	0.5	2.0	104.2(7.3)	96.3(7.1)	95.4(6.3)
Cadusafos	0.9928	0.3	1.0	103.5(9.5)	78.4(9.1)	80.2(7.4)	0.9964	0.3	1.0	114.1(9.3)	113.5(9.1)	92.2(7.3)
Tebutam	0.9949	3.0	10.0	110.3(8.1)	97.2(9.4)	99.3(6.4)	0.9949	3.0	10.0	92.9(9.2)	113.1(8.2)	96.3(7.3)

Promecarb	0.9991	1.0	3.0	80.2(9.2)	72.8(9.2)	92.1(9.1)	0.9982	1.0	3.0	102.3(4.2)	121.3(9.2)	99.3(8.3)
Phorate	0.9914	0.5	2.0	95.2(6.3)	90.4(9.5)	88.6(8.2)	0.9997	0.5	2.0	87.8(4.4)	97.9(6.3)	77.6(11.3)
Atratone	0.9950	3.0	10.0	95.5(8.6)	92.3(8.5)	100.5(5.2)	0.9944	3.0	10.0	89.6(7.5)	111.4(6.3)	95.5(9.0)
3,4,5-Trimethacarb	0.9925	1.0	3.0	81.4(4.4)	93.5(8.6)	80.4(9.8)	0.9987	3.0	10.0	95.4(6.2)	110.3(4.6)	86.7(6.8)
Dicloran	0.9935	3.0	10.0	95.1(6.3)	97.2(7.4)	99.3(9.1)	0.9911	3.0	10.0	92.7(7.8)	113.1(6.3)	96.5(8.6)
Pentachloroanisole	0.9992	3.0	10.0	78.8(9.6)	78.4(6.3)	84.5(6.4)	0.9910	3.0	10.0	81.6(6.7)	82.5(8.2)	82.8(7.2)
Ethoxyquin	0.9958	3.0	10.0	75.5(9.2)	73.4(8.9)	74.4(7.5)	0.9997	3.0	10.0	75.1(7.4)	78.5(8.2)	78.1(8.2)
Prometon	0.9991	1.0	3.0	82.8(5.1)	89.3(2.2)	92.3(6.2)	0.9945	3.0	10.0	83.3(3.1)	82.4(5.2)	89.2(5.3)
Atrazine	0.9945	1.0	3.0	88.2(8.4)	82.3(9.3)	95.3(7.1)	0.9940	1.0	3.0	103.4(7.1)	81.6(6.6)	93.2(6.1)
Monolinuron	0.9904	0.3	1.0	92.5(6.6)	93.5(5.4)	86.7(7.5)	0.9929	0.3	1.0	103.2(6.2)	84.5(6.4)	95.3(4.5)
Propazine	0.9910	0.3	1.0	105.5(7.3)	105.2(4.4)	94.5(5.3)	0.9946	0.3	1.0	79.8(8.2)	88.4(7.1)	98.5(7.2)
Clomazone	0.9935	0.3	1.0	95.3(6.1)	95.6(4.4)	94.5(4.6)	0.9936	0.3	1.0	105.6(8.1)	88.2(6.4)	88.5(7.2)
α-Hexachlorocyclohexane	0.9976	1.0	3.0	96.5(5.4)	80.3(4.3)	102.4(8.2)	0.9949	1.0	3.0	95.4(6.7)	108.5(9.6)	109.5(9.6)
Terbumeton	0.9970	0.3	1.0	102.3(7.4)	87.5(7.4)	101.2(5.5)	0.9951	0.3	1.0	92.5(6.2)	93.4(6.4)	103.1(8.3)
β-Hexachlorocyclohexane	0.9937	0.5	2.0	93.2(9.2)	84.1(6.2)	81.2(7.3)	0.9923	0.5	2.0	85.3(7.2)	98.3(8.4)	93.2(7.3)
Aminocarb	0.9920	0.5	2.0	106.1(8.3)	106.1(6.4)	94.3(5.1)	0.9972	0.5	2.0	95.8(6.2)	87.3(6.4)	88.1(9.2)
Isocarbamid	0.9993	0.3	1.0	104.5(7.1)	89.4(5.2)	91.6(6.3)	0.9938	0.5	2.0	103.3(9.5)	102.1(6.3)	93.5(8.3)
Cyromazine	0.9945	1.0	3.0	78.2(6.3)	87.5(7.2)	101.4(5.2)	0.9965	3.0	10.0	76.7(6.2)	93.2(6.1)	103.5(8.4)
γ-Hexachlorocyclohexane	0.9934	0.3	1.0	101.3(7.2)	108.3(9.2)	82.5(8.2)	0.9936	0.3	1.0	79.9(9.6)	80.1(9.4)	86.9(5.3)
Propetamphos	0.9984	0.3	1.0	105.3(8.2)	76.8(10.1)	80.5(9.3)	0.9969	0.3	1.0	103.5(9.2)	95.2(6.1)	93.4(8.2)
Cycluron	0.9933	0.3	1.0	94.5(7.1)	112.6(8.2)	108.6(5.1)	0.9968	0.5	2.0	95.4(6.4)	98.4(8.2)	97.7(5.3)
Terbuthylazine	0.9919	1.0	3.0	108.5(7.1)	87.4(7.3)	97.5(8.1)	0.9953	1.0	3.0	91.5(4.9)	92.6(6.2)	105.3(8.1)

Terbufos	0.9915	1.0	3.0	101.2(6.2)	79.1(9.1)	96.7(7.2)	0.9943	1.0	3.0	107.4(9.6)	105.5(7.1)	102.2(7.2)
Cyanophos	0.9990	0.5	2.0	96.2(10.8)	83.3(9.1)	80.7(9.1)	0.9987	0.5	2.0	94.8(8.4)	96.4(6.2)	93.1(7.2)
Trietazine	0.9970	1.0	3.0	104.1(7.3)	80.2(8.7)	95.8(6.2)	0.9967	1.0	3.0	108.5(6.3)	102.5(9.1)	109.3(7.2)
Quintozene	0.9967	3.0	10.0	75.4(9.1)	78.0(6.2)	82.3(5.8)	0.9955	3.0	10.0	73.5(6.2)	81.4(9.2)	82.1(7.1)
Fonofos	0.9916	3.0	10.0	73.8(6.1)	74.2(9.3)	74.1(6.3)	0.9981	3.0	10.0	75.8(6.2)	79.4(10.2)	79.1(9.5)
Pyroquilon	0.9983	1.0	3.0	74.2(8.2)	78.3(8.2)	84.3(5.2)	0.9998	3.0	10.0	74.3(9.1)	76.4(7.2)	79.7(4.3)
Dinoterb	0.9914	1.0	3.0	78.2(7.2)	83.1(9.3)	96.2(7.0)	0.9965	1.0	3.0	102.2(8.1)	81.8(6.8)	88.4(6.1)
Pyrimethanil	0.9996	0.3	1.0	122.2(6.9)	122.1(4.2)	120.3(7.1)	0.9926	0.3	1.0	120.4(6.2)	121.2(5.2)	120.5(4.0)
Diazinon	0.9956	0.3	1.0	103.5(6.1)	104.1(4.0)	94.4(5.3)	0.9929	0.3	1.0	79.8(9.2)	88.4(5.1)	97.2(6.3)
Flufenoxuron	0.9945	0.3	1.0	95.4(6.1)	95.3(4.3)	94.5(4.6)	0.9930	0.3	1.0	104.1(5.1)	88.8(6.4)	88.5(7.4)
Disulfoton	0.9903	0.3	1.0	102.3(3.2)	87.4(5.3)	101.5(5.1)	0.9946	0.3	1.0	92.2(6.4)	93.1(6.7)	103.4(6.2)
Paraoxon-methyl	0.9905	0.5	2.0	94.8(9.2)	84.3(2.4)	81.5(7.4)	0.9912	0.5	2.0	87.2(7.2)	99.1(8.3)	94.3(6.2)
Secbumeton	0.9955	0.5	2.0	105.2(8.1)	106.2(6.5)	95.2(3.1)	0.9935	0.5	2.0	95.4(5.1)	87.6(3.3)	88.4(9.5)
Aziprotryne	0.9906	0.3	1.0	104.3(5.1)	89.4(4.2)	91.7(4.3)	0.9966	0.5	2.0	103.1(2.0)	102.5(4.2)	93.1(8.2)
Dinitramine	0.9989	1.0	3.0	74.2(6.4)	87.2(7.4)	101.4(5.3)	0.9964	3.0	10.0	79.3(4.2)	93.4(5.1)	103.3(8.1)
Fenfuram	0.9990	0.3	1.0	101.4(6.1)	109.4(9.1)	82.55(8.1)	0.9983	0.3	1.0	79.5(9.4)	80.5(8.7)	86.3(5.5)
δ-Hexachlorocyclohexane	0.9963	0.3	1.0	105.3(5.1)	76.5(8.1)	81.0(8.3)	0.9903	0.3	1.0	102.1(9.3)	95.5(6.2)	93.2(9.2)
Mexacarbate	0.9936	0.3	1.0	94.5(7.2)	110.2(8.1)	107.2(5.2)	0.9923	0.5	2.0	95.1(6.3)	98.4(7.2)	97.2(5.2)
Isazofos	0.9957	1.0	3.0	108.1(7.5)	87.3(7.4)	97.2(6.1)	0.9936	1.0	3.0	91.4(4.5)	92.3(6.5)	105.1(7.1)
Chlorothalonil	0.9946	1.0	3.0	103.3(5.2)	79.2(9.1)	96.5(7.3)	0.9905	1.0	3.0	106.3(8.3)	104.1(7.2)	102.1(5.2)
Triallate	0.9988	0.5	2.0	96.5(5.5)	83.4(9.2)	80.2(9.2)	0.9932	0.5	2.0	94.1(8.3)	95.3(6.1)	93.4(9.3)
Tebupirimfos	0.9983	0.3	1.0	105.3(7.4)	80.5(9.6)	95.4(5.2)	0.9982	1.0	3.0	108.6(5.3)	102.2(5.1)	109.2(7.3)
musk ambrette	0.9955	0.3	1.0	106.4(7.4)	85.5(9.3)	96.4(5.5)	0.9960	0.3	1.0	79.4(10.1)	103.1(7.3)	104.1(7.2)
Oxabetrinil	0.9954	1.0	3.0	95.1(4.3)	93.2(9.1)	106.3(9.1)	0.9902	1.0	3.0	83.4(9.2)	79.4(9.3)	94.5(6.4)

Iprobenfos	0.9914	0.5	2.0	89.1(8.2)	74.3(9.4)	96.2(8.3)	0.9998	0.5	2.0	82.8(8.9)	105.0(9.1)	102.5(9.6)
Fluroxypyr	0.9968	0.5	2.0	92.4(6.5)	75.6(9.7)	107.5(6.6)	0.9967	0.5	2.0	84.1(7.2)	94.3(4.4)	84.8(6.9)
Pirimicarb	0.9947	0.5	2.0	85.0(7.1)	107.2(9.3)	117.2(9.1)	0.9966	0.5	2.0	93.7(9.8)	118.1(8.4)	123.4(9.5)
Monalide	0.9992	0.5	2.0	95.4(6.1)	80.2(9.4)	92.1(9.2)	0.9934	0.5	2.0	111.6(7.7)	103.8(8.9)	105.1(7.4)
Furmecyclox	0.9981	1.0	3.0	94.3(7.4)	73.5(9.6)	107.4(8.5)	0.9920	10	3.0	97.0(9.1)	94.2(9.3)	84.7(8.2)
Benoxacor	0.9947	1.0	3.0	84.6(8.3)	75.8(9.2)	106.6(8.7)	0.9995	3.0	10.0	101.3(5.0)	84.4(9.5)	94.0(8.1)
Pentachloroaniline	0.9913	0.3	1.0	92.5(8.4)	99.5(9.1)	96.9(7.2)	0.9960	0.3	1.0	85.5(4.6)	90.7(8.8)	105.2(4.3)
Benfuresate	0.9950	0.5	2.0	105.3(7.1)	81.2(9.5)	98.6(7.7)	0.9974	0.5	2.0	97.5(9.2)	103.4(8.5)	106.5(7.4)
Dioxacarb	0.9945	1.0	3.0	92.5(6.6)	90.4(4.2)	114.5(5.2)	0.9963	3.0	10.0	111.6(9.2)	112.3(6.1)	94.5(5.3)
Cyprazine	0.9957	0.3	1.0	103.4(6.1)	105.2(4.5)	109.2(6.3)	0.9927	0.3	1.0	108.4(4.3)	106.2(5.3)	106.9(6.2)
Phosphamidon	0.9931	3.0	10.0	103.4(4.5)	75.6(9.7)	107.9(8.1)	0.9973	3.0	10.0	94.5(6.6)	83.7(9.8)	84.2(8.3)
Dichlorprop	0.9917	0.3	1.0	104.2(7.3)	87.4(8.2)	104.5(7.1)	0.9969	0.3	1.0	84.5(8.7)	95.3(8.2)	101.4(5.3)
Dichlofenthion	0.9941	0.5	2.0	109.5(7.6)	92.7(4.8)	96.9(8.0)	0.9941	0.5	2.0	87.5(4.6)	104.7(5.8)	93.3(8.2)
Fenthion	0.9970	1.0	3.0	97.5(9.5)	84.6(9.7)	93.1(7.3)	0.9978	1.0	3.0	83.5(4.2)	103.3(7.3)	108.8(9.3)
Propanil	0.9970	0.3	1.0	101.1(4.3)	94.4(6.3)	103.1(4.2)	0.9958	0.5	2.0	102.3(9.2)	94.4(7.2)	95.5(4.2)
2,4-DB	0.9901	1.0	3.0	87.3(4.4)	84.5(8.1)	104.5(7.2)	0.9939	1.0	3.0	96.4(7.1)	92.5(8.3)	107.3(5.2)
Chlorthiamid	0.9909	0.5	2.0	94.2(8.2)	97.1(4.4)	113.4(7.2)	0.9936	0.5	2.0	98.2(7.3)	95.8(6.6)	108.6(8.3)
Dimethachlor	0.9932	0.3	1.0	106.5(4.4)	83.5(5.3)	97.3(9.1)	0.9917	0.3	1.0	78.5(9.2)	104.1(4.4)	109.4(2.1)
Metribuzin	0.9957	0.5	2.0	92.3(5.2)	102.4(9.4)	101.3(4.4)	0.9906	0.5	2.0	97.5(9.2)	92.5(8.4)	95.3(6.4)
Dimethenamid	0.9958	0.3	1.0	107.5(8.4)	96.5(7.1)	88.2(4.5)	0.9989	0.3	1.0	89.7(9.1)	103.3(9.5)	88.3(4.2)
Bromobutide	0.9965	0.5	2.0	96.3(7.4)	98.4(7.2)	94.2(8.2)	0.9946	0.5	2.0	104.2(7.3)	96.3(7.1)	95.4(6.3)
Terbucarb	0.9912	0.3	1.0	102.5(9.5)	78.4(9.1)	80.2(7.4)	0.9948	0.3	1.0	114.1(6.4)	113.5(9.1)	92.2(7.3)
Malaoxon	0.9925	0.5	2.0	110.3(8.1)	97.2(9.4)	99.3(6.4)	0.9953	3.0	10.0	92.9(9.2)	113.1(8.2)	96.3(7.3)
Vinclozolin	0.9923	1.0	3.0	86.2(9.2)	72.8(9.2)	92.1(9.1)	0.9991	1.0	3.0	101.3(4.2)	122.3(9.2)	99.3(8.3)
Parathion-methyl	0.9995	0.5	2.0	95.1(6.3)	90.4(9.5)	88.6(8.2)	0.9965	0.5	2.0	88.8(4.4)	97.9(6.3)	77.6(10.3)

Chlorpyrifos-methyl	0.9953	1.0	3.0	96.5(5.4)	80.3(4.3)	102.4(8.2)	0.9989	1.0	3.0	95.4(6.7)	108.5(9.6)	109.5(9.6)
Transfluthrin	0.9952	3.0	10.0	95.5(7.6)	92.3(8.5)	100.5(5.2)	0.9980	3.0	10.0	88.6(7.5)	111.4(6.3)	95.5(9.0)
Simetryn	0.9901	1.0	3.0	81.4(8.4)	93.5(8.6)	80.4(9.8)	0.9969	3.0	10.0	95.4(6.2)	110.3(5.6)	86.7(6.8)
Fuberidazole	0.9941	3.0	10.0	95.1(6.3)	97.2(7.4)	99.3(9.1)	0.9973	3.0	10.0	92.7(7.8)	113.1(8.3)	96.5(8.6)
Tolclofos-methyl	0.9948	1.0	3.0	78.6(9.6)	78.4(6.3)	84.5(6.4)	0.9959	3.0	10.0	79.6(6.7)	82.5(9.2)	82.8(7.2)
Alachlor	0.9980	3.0	10.0	75.5(9.2)	79.2(8.9)	74.4(10.4)	0.9955	3.0	10.0	75.1(7.4)	78.5(8.2)	78.1(8.2)
Ametryn	0.9939	1.0	3.0	82.7(5.1)	89.3(6.2)	92.3(6.2)	0.9905	3.0	10.0	85.3(3.1)	82.4(5.2)	89.2(5.3)
Heptachlor	0.9953	1.0	3.0	88.2(8.4)	82.3(9.3)	95.3(7.1)	0.9908	1.0	3.0	103.4(7.1)	81.6(6.8)	93.2(6.1)
Prometryn	0.9922	0.3	1.0	92.5(5.6)	93.5(5.4)	86.7(7.5)	0.9969	0.3	1.0	103.2(6.2)	84.5(6.5)	95.3(4.5)
Acetochlor	0.9950	0.3	1.0	105.5(7.3)	105.2(4.4)	94.5(5.3)	0.9939	0.3	1.0	79.8(8.2)	88.4(7.1)	98.5(7.2)
Paraoxon-ethyl	0.9997	0.3	1.0	95.3(6.1)	95.6(4.4)	94.5(4.6)	0.9956	0.3	1.0	105.6(8.1)	88.2(6.4)	88.5(7.2)
Metalaxyl	0.9908	0.3	1.0	102.3(8.4)	87.5(7.4)	101.2(5.5)	0.9927	0.3	1.0	92.5(6.2)	93.4(6.4)	103.1(8.3)
Tridiphane	0.9906	0.5	2.0	93.9(9.2)	84.1(6.2)	81.2(7.3)	0.9993	0.5	2.0	85.3(7.2)	99.3(8.4)	93.2(7.3)
Octachlorodipropyl ether	0.9984	0.5	2.0	106.7(8.3)	106.1(6.4)	94.3(5.1)	0.9996	0.5	2.0	95.2(6.2)	87.3(6.4)	88.1(9.2)
Prosulfocarb	0.9973	0.3	1.0	104.5(7.1)	89.4(5.2)	91.4(6.3)	0.9950	0.5	2.0	103.3(9.3)	102.1(6.3)	93.5(8.3)
Fenpropidin	0.9906	1.0	3.0	78.2(6.3)	87.5(7.2)	101.4(5.2)	0.9921	3.0	10.0	82.9(6.2)	93.2(6.1)	103.5(8.4)
1-Naphthylacetamide	0.9988	0.3	1.0	101.3(6.2)	108.3(9.2)	82.5(8.2)	0.9941	0.3	1.0	79.9(9.7)	80.1(9.4)	86.9(5.3)
Dithiopyr	0.9984	0.3	1.0	105.3(8.2)	76.8(9.1)	80.5(9.3)	0.9922	0.3	1.0	103.5(9.2)	95.2(6.1)	93.4(8.2)
Orbencarb	0.9988	0.3	1.0	94.5(9.1)	112.6(8.2)	108.6(5.1)	0.9955	0.5	2.0	95.4(6.2)	98.5(8.2)	97.7(5.3)
Terbutryn	0.9932	1.0	3.0	108.5(7.1)	87.4(7.3)	97.5(8.1)	0.9951	1.0	3.0	91.5(4.9)	92.6(6.2)	105.3(8.1)
Spiroxamine	0.9957	1.0	3.0	101.6(6.2)	79.1(9.1)	96.7(7.2)	0.9968	1.0	3.0	107.4(9.3)	105.5(7.1)	102.2(7.2)
Methiocarb	0.9948	0.5	2.0	96.2(5.2)	83.3(9.1)	80.7(9.1)	0.9973	0.5	2.0	94.8(8.4)	95.4(6.2)	93.1(7.2)
Fenitrothion	0.9967	1.0	3.0	102.1(7.3)	80.2(8.7)	95.8(6.2)	0.9984	1.0	3.0	108.5(6.3)	102.5(9.1)	109.3(7.2)
Pirimiphos-methyl	0.9935	0.3	1.0	106.8(7.3)	85.1(9.2)	96.9(5.4)	0.9973	0.3	1.0	79.9(9.2)	103.8(7.2)	104.2(7.1)

Methiocarb sulfone	0.9992	3.0	10.0	78.4(9.1)	78.0(6.2)	82.3(5.4)	0.9967	3.0	10.0	74.5(5.2)	81.4(7.2)	82.1(7.1)
Ethofumesate	0.9993	3.0	10.0	79.6(7.1)	74.2(9.3)	74.1(6.5)	0.9910	3.0	10.0	75.8(6.2)	79.4(10.2)	79.1(9.5)
Linuron	0.9999	1.0	3.0	73.2(9.2)	78.3(8.2)	84.3(5.3)	0.9961	3.0	10.0	74.3(9.1)	76.4(7.2)	79.7(4.3)
Probenazole	0.9969	1.0	3.0	78.2(7.2)	83.1(9.3)	96.2(7.1)	0.9902	1.0	3.0	102.2(8.1)	81.8(6.8)	88.4(6.1)
Noruron	0.9972	0.3	1.0	91.2(6.4)	92.1(4.2)	86.3(7.4)	0.9920	0.3	1.0	103.4(6.2)	82.2(5.2)	95.5(4.0)
Quinoclamine	0.9926	0.3	1.0	103.8(7.1)	104.1(4.0)	94.4(5.6)	0.9939	0.3	1.0	79.8(9.4)	88.4(5.1)	97.2(6.3)
Dipropetryn	0.9902	0.3	1.0	95.4(6.1)	95.3(4.3)	94.5(2.4)	0.9955	0.3	1.0	105.1(5.1)	87.8(6.4)	88.5(7.4)
Malathion	0.9979	0.3	1.0	102.4(6.2)	87.4(5.3)	101.5(5.6)	1.0000	0.3	1.0	92.2(6.6)	93.1(6.7)	103.4(6.2)
Thiobencarb	0.9903	0.5	2.0	94.3(9.2)	84.3(5.4)	81.5(7.7)	0.9969	0.5	2.0	87.2(7.2)	99.1(8.3)	94.3(6.2)
Diethofencarb	0.9904	0.5	2.0	107.2(8.1)	106.2(6.5)	95.2(3.9)	0.9927	0.5	2.0	95.4(5.1)	87.6(5.1)	88.4(9.5)
Phorate sulfoxide	0.9925	0.3	1.0	104.3(5.1)	89.4(4.2)	91.7(5.3)	0.9937	0.3	1.0	103.1(6.1)	101.5(4.6)	93.1(8.2)
Metolachlor	0.9986	1.0	3.0	74.2(6.4)	87.2(7.4)	101.4(4.3)	0.9991	3.0	10.0	76.4(4.2)	93.4(4.1)	103.3(8.1)
Fenpropimorph	0.9916	0.3	1.0	101.4(6.1)	109.4(9.1)	82.55(6.1)	0.9908	0.3	1.0	79.5(9.4)	80.5(7.7)	86.3(5.5)
Cyanazine	0.9935	0.3	1.0	105.3(8.1)	76.5(8.1)	81.0(7.3)	0.9945	0.3	1.0	102.1(9.3)	95.5(6.1)	93.2(9.2)
Chlorpyrifos	0.9989	0.3	1.0	94.5(7.2)	110.2(8.1)	107.2(5.8)	0.9985	0.5	2.0	95.1(6.3)	98.4(7.5)	97.2(5.2)
Parathion	0.9942	1.0	3.0	107.1(7.5)	87.3(7.4)	97.2(6.2)	0.9904	1.0	3.0	91.4(4.5)	92.3(6.3)	105.1(7.1)
Flufenacet	0.9978	1.0	3.0	101.3(5.2)	79.2(9.1)	96.5(7.8)	0.9981	1.0	3.0	106.6(8.3)	104.1(7.6)	102.1(5.2)
Rabenzazol	0.9941	0.5	2.0	96.5(5.5)	83.4(9.2)	80.2(9.1)	0.9991	0.5	2.0	94.1(8.3)	95.3(6.5)	93.4(9.3)
4,4'-Dichlorobenzophenone	0.9902	1.0	3.0	104.3(7.4)	80.5(9.6)	95.4(5.2)	0.9951	1.0	3.0	108.6(5.7)	102.2(5.1)	109.2(7.3)
Triadimefon	0.9927	0.3	1.0	107.4(7.4)	85.5(9.3)	96.4(5.5)	0.9901	0.3	1.0	79.4(10.1)	103.1(7.3)	104.1(7.2)
Chlorthal-dimethyl	0.9945	1.0	3.0	95.1(6.3)	93.2(9.1)	106.3(9.1)	0.9955	1.0	3.0	83.4(9.1)	79.4(9.3)	94.5(6.4)
Dicapthon	0.9980	0.5	2.0	89.1(8.2)	74.3(9.4)	96.2(8.3)	0.9967	0.5	2.0	82.8(8.6)	1054(9.1)	102.5(9.6)
Isofenphos-oxon	0.9939	0.5	2.0	92.4(6.5)	75.6(9.7)	107.5(6.6)	0.9968	0.5	2.0	84.1(7.2)	94.3(4.4)	84.8(6.9)
Isocarbophos	0.9924	0.5	2.0	85.0(7.1)	107.2(9.3)	117.2(7.1)	0.9929	0.5	2.0	93.7(9.2)	118.1(8.4)	123.4(9.5)

Tetraconazole	0.9939	0.5	2.0	95.4(6.1)	80.2(9.4)	92.1(9.2)	0.9945	0.5	2.0	111.6(7.7)	103.8(8.9)	105.1(7.4)
Isobenzan	0.9973	1.0	3.0	94.3(9.4)	79.5(9.6)	107.4(8.5)	0.9990	10	3.0	97.2(9.1)	94.2(9.3)	84.7(8.2)
Flurochloridone	0.9943	1.0	3.0	84.6(7.3)	75.8(9.2)	106.6(7.7)	0.9903	3.0	10.0	101.3(5.3)	84.4(9.5)	94.0(8.1)
Fenson	0.9976	0.3	1.0	90.5(8.4)	99.5(9.1)	96.9(7.2)	0.9971	0.3	1.0	85.5(4.6)	90.7(8.8)	105.2(4.3)
Pyracarbolid	0.9949	0.5	2.0	105.3(7.1)	81.2(9.5)	98.6(7.7)	0.9995	0.5	2.0	97.5(9.2)	103.4(8.5)	106.5(7.4)
Dodemorph	0.9925	1.0	3.0	92.5(6.6)	90.4(4.2)	94.5(5.2)	0.9911	3.0	10.0	81.6(9.5)	92.3(6.1)	94.5(5.3)
Mgk 264	0.9961	0.3	1.0	93.4(6.1)	94.2(4.5)	100.2(6.3)	0.9949	0.3	1.0	90.4(4.3)	97.2(5.3)	96.9(6.2)
Butralin	0.9909	3.0	10.0	103.4(4.5)	75.6(9.7)	107.9(8.1)	0.9988	3.0	10.0	94.5(6.6)	83.7(8.8)	84.2(8.3)
Carbaryl	0.9940	0.3	1.0	104.2(7.3)	87.4(8.2)	104.5(7.1)	0.9931	0.3	1.0	84.5(8.4)	95.3(8.2)	101.4(5.3)
Diphenamid	0.9958	0.5	2.0	109.5(7.6)	92.7(4.8)	96.9(8.0)	0.9960	0.5	2.0	87.5(4.6)	104.7(5.8)	93.3(8.2)
Pirimiphos-ethyl	0.9997	1.0	3.0	97.4(9.5)	84.6(9.7)	93.1(7.3)	0.9960	1.0	3.0	83.5(4.2)	103.3(7.3)	108.8(9.3)
Isodrin	0.9908	0.3	1.0	101.1(4.3)	94.4(6.3)	103.1(4.2)	0.9990	0.5	2.0	102.3(9.6)	94.4(7.2)	95.5(4.2)
Aldrin	0.9915	1.0	3.0	85.3(4.4)	84.5(8.1)	105.5(7.2)	0.9990	1.0	3.0	96.4(7.1)	92.5(8.3)	107.3(5.2)
Isopropalin	0.9983	0.5	2.0	94.2(8.2)	97.1(4.4)	113.4(7.2)	0.9976	0.5	2.0	98.2(7.3)	95.8(6.6)	108.6(8.3)
Cyprodinil	0.9953	0.3	1.0	104.5(4.4)	83.5(5.3)	97.3(9.1)	0.9931	0.3	1.0	78.5(7.2)	104.1(4.4)	109.4(9.5)
Isofenphos-methyl	0.9913	0.5	2.0	92.3(8.2)	102.4(9.4)	101.3(4.4)	0.9912	0.5	2.0	97.5(9.2)	92.5(8.4)	95.3(6.4)
Octachlorostyrene	0.9922	0.3	1.0	107.5(9.4)	96.5(7.1)	88.2(4.5)	0.9958	0.3	1.0	102.4(8.1)	103.3(8.5)	88.3(4.2)
Metazachlor	0.9930	0.5	2.0	92.3(7.4)	98.4(7.2)	94.2(8.2)	0.9979	0.5	2.0	104.2(7.3)	96.3(7.1)	95.4(6.3)
Dimethametryn	0.9960	0.3	1.0	103.5(9.5)	78.4(9.1)	80.2(7.4)	0.9906	0.3	1.0	114.1(6.4)	113.5(9.1)	92.2(7.3)
Pendimethalin	0.9924	3.0	10.0	110.3(8.1)	97.2(9.4)	99.3(6.4)	0.9963	3.0	10.0	92.9(9.2)	113.1(8.2)	96.3(7.3)
Disulfoton-sulfone	0.9962	1.0	3.0	80.2(9.2)	72.8(9.2)	92.1(9.1)	0.9926	1.0	3.0	102.3(4.2)	121.3(9.2)	99.3(8.3)
Phorate sulfone	0.9995	0.5	2.0	95.2(6.3)	90.4(9.5)	88.6(8.2)	0.9998	0.5	2.0	87.8(4.4)	97.9(6.3)	77.6(10.4)
Terbufos sulfone	0.9939	1.0	3.0	96.5(5.4)	80.3(4.3)	102.4(8.2)	0.9948	1.0	3.0	95.4(6.7)	108.5(9.6)	109.5(9.6)
Paclobutrazol	0.9958	3.0	10.0	95.5(8.6)	92.3(8.5)	100.5(5.2)	0.9963	3.0	10.0	89.6(7.5)	111.4(6.3)	95.5(9.0)
Penconazole	0.9917	1.0	3.0	81.4(4.4)	93.5(8.6)	80.4(9.8)	0.9953	3.0	10.0	95.4(6.2)	110.3(4.6)	86.7(6.8)

Chlozolinate	0.9969	3.0	10.0	95.1(6.3)	97.2(7.4)	99.3(9.1)	0.9902	3.0	10.0	92.7(7.8)	113.1(6.3)	96.5(8.6)
Pyrifenox	0.9926	3.0	10.0	78.8(9.6)	78.4(6.3)	84.5(6.4)	0.9969	3.0	10.0	81.6(6.7)	82.5(8.2)	82.8(7.2)
Tolylfluanid	0.9909	3.0	10.0	115.5(9.2)	113.4(8.9)	104.4(7.5)	0.9915	3.0	10.0	105.1(7.4)	108.5(8.2)	108.1(8.2)
Fosthiazate	0.9906	1.0	3.0	82.8(5.1)	89.3(2.2)	92.3(6.2)	0.9916	3.0	10.0	83.3(3.1)	82.4(5.2)	89.2(5.3)
Phosfolan	0.9985	1.0	3.0	88.2(8.4)	82.3(9.3)	95.3(7.1)	0.9960	1.0	3.0	103.4(7.1)	81.6(6.6)	93.2(6.1)
Allethrin	0.9924	0.3	1.0	92.5(6.6)	93.5(5.4)	86.7(7.5)	0.9965	0.3	1.0	103.2(6.2)	84.5(6.4)	95.3(4.5)
Isofenphos	0.9924	0.3	1.0	105.5(7.3)	105.2(4.4)	94.5(5.3)	0.9918	0.3	1.0	79.8(8.2)	88.4(7.1)	98.5(7.2)
Captan	0.9999	0.3	1.0	95.3(6.1)	95.6(4.4)	94.5(4.6)	0.9963	0.3	1.0	105.6(8.1)	88.2(6.4)	88.5(7.2)
Fipronil	0.9985	0.3	1.0	102.3(7.4)	87.5(7.4)	101.2(5.5)	0.9916	0.3	1.0	92.5(6.2)	93.4(6.4)	103.1(8.3)
Diclocymet	0.9916	0.5	2.0	93.2(9.2)	84.1(6.2)	81.2(7.3)	0.9970	0.5	2.0	85.3(7.2)	98.3(8.4)	93.2(7.3)
Quinalphos	0.9908	0.5	2.0	106.1(8.3)	106.1(6.4)	94.3(5.1)	0.9981	0.5	2.0	95.8(6.2)	87.3(6.4)	88.1(9.2)
Phenthoate	0.9927	0.3	1.0	104.5(7.1)	89.4(5.2)	91.6(6.3)	0.9914	0.5	2.0	103.3(9.5)	102.1(6.3)	93.5(8.3)
Triadimenol	0.9951	1.0	3.0	78.2(6.3)	87.5(7.2)	101.4(5.2)	0.9904	3.0	10.0	72.8(6.2)	93.2(6.1)	103.5(8.4)
Dinobuton	0.9974	0.3	1.0	101.3(7.2)	108.3(9.2)	82.5(8.2)	0.9949	0.3	1.0	79.9(9.6)	80.1(9.4)	86.9(5.3)
Furalaxyl	0.9971	0.3	1.0	105.3(8.2)	76.8(9.1)	80.5(9.3)	0.9995	0.3	1.0	103.5(9.2)	95.2(6.1)	93.4(8.2)
Crotoxyphos	0.9992	0.3	1.0	94.5(7.1)	112.6(8.2)	108.6(5.1)	0.9941	0.5	2.0	95.4(6.4)	98.4(8.2)	97.7(5.3)
Procymidone	0.9975	1.0	3.0	108.5(7.1)	87.4(7.3)	97.5(8.1)	0.9915	1.0	3.0	91.5(4.9)	92.6(6.2)	105.3(8.1)
Chlorbenside	0.9966	1.0	3.0	101.2(6.2)	79.1(9.1)	96.7(7.2)	0.9962	1.0	3.0	107.4(9.6)	105.5(7.1)	102.2(7.2)
Chlorflurenol-methyl	0.9934	0.5	2.0	96.2(5.2)	83.3(9.1)	80.7(9.1)	0.9955	0.5	2.0	94.8(8.4)	96.4(6.2)	93.1(7.2)
Chlordane	0.9969	1.0	3.0	104.1(7.3)	80.2(8.7)	95.8(6.2)	0.9970	1.0	3.0	108.5(6.3)	102.5(9.1)	109.3(7.2)
Methidathion	0.9919	3.0	10.0	75.4(9.1)	78.0(6.2)	82.3(5.8)	0.9956	3.0	10.0	73.5(6.2)	81.4(9.2)	82.1(7.1)
Haloxyfop-methyl	0.9918	3.0	10.0	113.8(6.1)	114.2(9.3)	104.1(6.3)	0.9993	3.0	10.0	105.8(6.2)	109.4(10.2)	119.1(9.5)
Bromophos-ethyl	0.9900	1.0	3.0	74.2(8.2)	78.3(8.2)	84.3(5.2)	0.9970	3.0	10.0	74.3(9.1)	76.4(7.2)	79.7(4.3)
Procyazine	0.9904	1.0	3.0	78.2(7.2)	83.1(9.3)	96.2(7.0)	0.9932	1.0	3.0	102.2(8.1)	81.8(6.8)	88.4(6.1)
Disulfoton-sulfoxide	0.9908	0.3	1.0	94.2(6.9)	92.1(4.2)	86.3(7.1)	0.9909	0.3	1.0	103.4(6.2)	84.2(5.2)	95.5(4.0)

Tetrachlorvinphos	0.9974	0.3	1.0	103.5(6.1)	104.1(4.0)	94.4(5.3)	0.9991	0.3	1.0	79.8(9.2)	88.4(5.1)	97.2(6.3)
Endosulfan	0.9960	0.3	1.0	95.4(6.1)	95.3(4.3)	94.5(4.6)	0.9943	0.3	1.0	104.1(5.1)	88.8(6.4)	88.5(7.4)
Mepanipyrim	0.9990	0.3	1.0	102.3(3.2)	87.4(5.3)	101.5(5.1)	0.9968	0.3	1.0	92.2(6.4)	93.1(6.7)	103.4(6.2)
Butachlor	0.9950	0.5	2.0	94.8(9.2)	84.3(5.4)	81.5(7.4)	0.9985	0.5	2.0	87.2(7.2)	99.1(8.3)	94.3(6.2)
Ditalimfos	0.9952	0.5	2.0	105.2(8.1)	106.2(6.5)	95.2(3.1)	0.9935	0.5	2.0	95.4(5.1)	87.6(3.3)	88.4(9.5)
TCMTB	0.9986	0.3	1.0	104.3(5.1)	89.4(4.2)	91.7(4.3)	0.9943	0.5	2.0	103.1(6.1)	102.5(4.2)	93.1(8.2)
trans-Nonachlor	0.9912	1.0	3.0	114.2(6.4)	117.2(7.4)	101.4(5.3)	0.9955	3.0	10.0	112.8(4.2)	113.4(5.1)	113.3(8.1)
Chlorfenson	0.9992	0.3	1.0	101.4(6.1)	109.4(9.1)	82.55(8.1)	0.9973	0.3	1.0	79.5(9.4)	80.5(8.7)	86.3(5.5)
Fenamiphos	0.9912	0.3	1.0	105.3(5.1)	76.5(8.1)	81.0(8.3)	0.9924	0.3	1.0	102.1(9.3)	95.5(6.2)	93.2(9.2)
Picoxystrobin	0.9919	0.3	1.0	94.5(7.2)	110.2(8.1)	107.2(5.2)	0.9912	0.5	2.0	95.1(6.3)	98.4(7.2)	97.2(5.2)
Napropamide	0.9972	1.0	3.0	108.1(7.5)	87.3(7.4)	97.2(6.1)	1.0000	1.0	3.0	91.4(4.5)	92.3(6.5)	105.1(7.1)
Hexaconazole	0.9943	1.0	3.0	103.3(5.2)	79.2(9.1)	96.5(7.3)	0.9933	1.0	3.0	106.3(8.3)	104.1(7.2)	102.1(5.2)
Flutolanil	0.9906	0.5	2.0	96.5(5.5)	83.4(9.2)	80.2(9.2)	0.9922	0.5	2.0	94.1(8.3)	95.3(6.1)	93.4(9.3)
Prothiophos	0.9928	0.3	1.0	105.3(7.4)	80.5(9.6)	95.4(5.2)	0.9933	1.0	3.0	108.6(5.3)	102.2(5.1)	109.2(7.3)
Isoprothiolane	0.9914	0.3	1.0	106.4(7.4)	85.5(9.3)	96.4(5.5)	0.9928	0.3	1.0	79.4(10.1)	103.1(7.3)	104.1(7.2)
Profenofos	0.9914	1.0	3.0	95.1(4.3)	93.2(9.1)	106.3(9.1)	0.9936	1.0	3.0	83.4(9.2)	79.4(9.3)	94.5(6.4)
tricyclazole	0.9906	0.5	2.0	89.1(8.2)	74.3(9.4)	96.2(8.3)	0.9963	0.5	2.0	82.8(8.9)	105.0(9.1)	102.5(9.6)
Pretilachlor	0.9986	0.5	2.0	92.4(6.5)	75.6(9.7)	107.5(6.6)	0.9916	0.5	2.0	84.1(7.2)	94.3(4.4)	84.8(6.9)
Dieldrin	0.9965	0.5	2.0	85.0(7.1)	107.2(9.3)	117.2(9.1)	0.9968	0.5	2.0	93.7(9.8)	118.1(8.4)	123.4(9.5)
Oxadiazon	0.9982	0.5	2.0	95.4(6.1)	80.2(9.4)	92.1(9.2)	0.9922	0.5	2.0	111.6(7.7)	103.8(8.9)	105.1(7.4)
Iprovalicarb	0.9984	1.0	3.0	94.3(7.4)	73.5(9.6)	107.4(8.5)	0.9970	10	3.0	97.0(9.1)	94.2(9.3)	84.7(8.2)
Carboxin	0.9922	1.0	3.0	84.6(8.3)	75.8(9.2)	106.6(8.7)	0.9938	3.0	10.0	101.3(5.0)	84.4(9.5)	94.0(8.1)
Myclobutanil	0.9934	0.3	1.0	122.5(8.4)	121.5(9.1)	121.9(7.2)	0.9980	0.3	1.0	123.5(4.6)	120.7(8.8)	122.2(4.3)
p,p'-Dichlorodiphenyldichloroethylene	0.9951	0.5	2.0	105.3(7.1)	81.2(9.5)	98.6(7.7)	0.9958	0.5	2.0	97.5(9.2)	103.4(8.5)	106.5(7.4)

Buprofezin	0.9923	1.0	3.0	92.5(6.6)	90.4(4.2)	94.5(5.2)	0.9920	3.0	10.0	81.6(9.2)	92.3(6.1)	94.5(5.3)
Imazalil	0.9965	0.3	1.0	93.4(6.1)	94.2(4.5)	100.2(6.3)	0.9994	0.3	1.0	90.4(4.3)	97.2(5.3)	96.9(6.2)
Flusilazole	0.9995	3.0	10.0	103.4(4.5)	75.6(9.7)	107.9(8.1)	0.9955	3.0	10.0	94.5(6.6)	83.7(9.8)	84.2(8.3)
Methoprotryne	0.9962	0.3	1.0	104.2(7.3)	87.4(8.2)	104.5(7.1)	0.9971	0.3	1.0	84.5(8.7)	95.3(8.2)	101.4(5.3)
Azaconazole	0.9978	0.5	2.0	109.5(7.6)	92.7(4.8)	96.9(8.0)	0.9914	0.5	2.0	87.5(4.6)	104.7(5.8)	93.3(8.2)
Bupirimate	0.9969	1.0	3.0	97.5(9.5)	84.6(9.7)	93.1(7.3)	0.9919	1.0	3.0	83.5(4.2)	103.3(7.3)	108.8(9.3)
Imazamethabenz-methyl	0.9939	0.3	1.0	101.1(4.3)	94.4(6.3)	103.1(4.2)	0.9906	0.5	2.0	102.3(9.2)	94.4(7.2)	95.5(4.2)
Kresoxim-methyl	0.9956	1.0	3.0	87.3(4.4)	84.5(8.1)	104.5(7.2)	0.9994	1.0	3.0	96.4(7.1)	92.5(8.3)	107.3(5.2)
Metamitron	0.9999	0.5	2.0	94.2(8.2)	97.1(4.4)	113.4(7.2)	0.9989	0.5	2.0	98.2(7.3)	95.8(6.6)	108.6(8.3)
Isoxathion	0.9921	0.3	1.0	106.5(4.4)	83.5(5.3)	97.3(9.1)	0.9960	0.3	1.0	78.5(9.2)	104.1(4.4)	109.4(9.5)
Aramite	0.9942	0.5	2.0	92.3(5.2)	102.4(9.4)	101.3(4.4)	0.9995	0.5	2.0	97.5(9.2)	92.5(8.4)	95.3(6.4)
Nitrofen	0.9951	0.3	1.0	107.5(8.4)	96.5(7.1)	88.2(4.5)	0.9976	0.3	1.0	72.4(9.1)	103.3(9.5)	88.3(4.2)
Endrin	0.9926	0.5	2.0	96.3(7.4)	98.4(7.2)	94.2(8.2)	0.9990	0.5	2.0	104.2(7.3)	96.3(7.1)	95.4(6.3)
Endrin aldehyde	0.9923	0.3	1.0	102.5(9.5)	78.4(9.1)	80.2(7.4)	0.9966	0.3	1.0	114.1(6.4)	113.5(9.1)	92.2(7.3)
Ancymidol	0.9919	0.5	2.0	110.3(8.1)	97.2(9.4)	99.3(6.4)	0.9902	3.0	10.0	92.9(9.2)	113.1(8.2)	96.3(7.3)
Perthan	0.9916	1.0	3.0	86.2(9.2)	72.8(9.2)	92.1(9.1)	0.9978	1.0	3.0	101.3(4.2)	122.3(9.2)	99.3(8.3)
Chlorfenapyr	0.9963	0.5	2.0	113.1(6.3)	110.4(9.5)	113.6(8.2)	0.9932	0.5	2.0	110.8(4.4)	112.9(6.3)	111.6(10.3)
Chloropropylate	0.9988	1.0	3.0	96.5(5.4)	80.3(4.3)	102.4(8.2)	0.9987	1.0	3.0	95.4(6.7)	108.5(9.6)	109.5(9.6)
Chlorobenzilate	0.9948	3.0	10.0	95.5(7.6)	92.3(8.5)	100.5(5.2)	0.9949	3.0	10.0	88.6(7.5)	111.4(6.3)	95.5(9.0)
Fenthion sulfoxide	0.9977	1.0	3.0	81.4(8.4)	93.5(8.6)	80.4(9.8)	0.9912	3.0	10.0	95.4(6.2)	110.3(5.6)	86.7(6.8)
Diniconazole	0.9952	3.0	10.0	95.1(6.3)	97.2(7.4)	99.3(9.1)	0.9951	3.0	10.0	92.7(7.8)	113.1(8.3)	96.5(8.6)
Flamprop-isopropyl	0.9950	1.0	3.0	108.6(9.6)	108.4(6.3)	104.5(6.4)	0.9975	3.0	10.0	101.6(6.7)	102.5(9.2)	102.8(7.2)
p,p'-Dichlorodiphenyldichloroethane	0.9919	3.0	10.0	75.5(9.2)	73.4(8.9)	74.4(7.5)	0.9999	3.0	10.0	75.1(7.4)	78.5(8.2)	78.1(8.2)

Aclonifen	0.9916	1.0	3.0	82.7(5.1)	89.3(6.2)	92.3(6.2)	0.9980	3.0	10.0	85.3(3.1)	82.4(5.2)	89.2(5.3)
o,p'-Dichlorodiphenyltri chloroethane	0.9965	1.0	3.0	88.2(8.4)	82.3(9.3)	95.3(7.1)	0.9967	1.0	3.0	103.4(7.1)	81.6(6.8)	93.2(6.1)
Oxadixyl	0.9914	0.3	1.0	92.5(5.6)	93.5(5.4)	86.7(7.5)	0.9999	0.3	1.0	103.2(6.2)	84.5(6.5)	95.3(4.5)
Ethion	0.9912	0.3	1.0	105.5(7.3)	105.2(4.4)	94.5(5.3)	0.9998	0.3	1.0	79.8(8.2)	88.4(7.1)	98.5(7.2)
Mepronil	0.9957	0.3	1.0	95.3(6.1)	95.6(4.4)	94.5(4.6)	0.9926	0.3	1.0	105.6(8.1)	88.2(6.4)	88.5(7.2)
Triazophos	0.9961	0.3	1.0	102.3(8.4)	87.5(7.4)	101.2(5.5)	0.9930	0.3	1.0	92.5(6.2)	93.4(6.4)	103.1(8.3)
Azamethiphos	0.9963	0.5	2.0	93.9(9.2)	84.1(6.2)	81.2(7.3)	0.9970	0.5	2.0	85.3(7.2)	99.3(8.4)	93.2(7.3)
Ofurace	0.9913	0.5	2.0	106.7(8.3)	106.1(6.4)	94.3(5.1)	0.9980	0.5	2.0	95.2(6.2)	87.3(6.4)	88.1(9.2)
Carbophenothion	0.9988	0.3	1.0	104.5(7.1)	89.4(5.2)	91.4(6.3)	0.9926	0.5	2.0	103.3(9.3)	102.1(6.3)	93.5(8.3)
Benalaxyl	0.9929	1.0	3.0	118.2(6.3)	117.5(7.2)	101.4(5.2)	0.9949	3.0	10.0	102.9(6.2)	103.2(6.1)	113.5(8.4)
Tepraloxydim	0.9914	0.3	1.0	101.3(6.2)	108.3(9.2)	82.5(8.2)	0.9999	0.3	1.0	79.9(9.7)	80.1(9.4)	86.9(5.3)
Diofenolan	0.9978	0.3	1.0	105.3(8.2)	76.8(9.1)	80.5(9.3)	0.9905	0.3	1.0	103.5(9.2)	95.2(6.1)	93.4(8.2)
Cyanofenphos	0.9987	0.3	1.0	94.5(9.1)	112.6(8.2)	108.6(5.1)	0.9975	0.5	2.0	95.4(6.2)	98.5(8.2)	97.7(5.3)
Edifenphos	0.9909	1.0	3.0	108.5(7.1)	87.4(7.3)	97.5(8.1)	0.9966	1.0	3.0	91.5(4.9)	92.6(6.2)	105.3(8.1)
Quinoxyfen	0.9954	1.0	3.0	101.6(6.2)	79.1(9.1)	96.7(7.2)	0.9990	1.0	3.0	107.4(9.3)	105.5(7.1)	102.2(7.2)
Endosulfan Sulfate	0.9999	0.5	2.0	96.2(5.2)	83.3(9.1)	80.7(9.1)	0.9968	0.5	2.0	94.8(8.4)	95.4(6.2)	93.1(7.2)
Propiconazol	0.9926	1.0	3.0	102.1(7.3)	80.2(8.7)	95.8(6.2)	0.9960	1.0	3.0	108.5(6.3)	102.5(9.1)	109.3(7.2)
Norflurazon	0.9997	3.0	10.0	108.4(9.1)	108.0(6.2)	102.3(5.4)	0.9971	3.0	10.0	104.5(5.2)	101.4(7.2)	102.1(7.1)
Fenhexamid	0.9934	3.0	10.0	103.4(7.1)	104.2(9.3)	104.1(6.5)	0.9994	3.0	10.0	105.8(6.2)	109.4(10.2)	109.1(9.5)
p,p'-Dichlorodiphenyltri chloroethane	0.9905	1.0	3.0	113.2(9.2)	118.3(8.2)	114.3(5.3)	0.9940	3.0	10.0	114.3(9.1)	116.4(7.2)	119.7(4.3)
Trifloxystrobin	0.9931	1.0	3.0	78.2(7.2)	83.1(9.3)	96.2(7.1)	0.9949	1.0	3.0	102.2(8.1)	81.8(6.8)	88.4(6.1)
Hexazinone	0.9978	0.3	1.0	91.2(6.4)	92.1(4.2)	86.3(7.4)	0.9925	0.3	1.0	103.4(6.2)	82.2(5.2)	95.5(4.0)
Tebuconazol	0.9946	0.3	1.0	103.8(7.1)	104.1(4.0)	94.4(5.6)	0.9948	0.3	1.0	79.8(9.4)	88.4(5.1)	97.2(6.3)

Chloridazon	0.9976	0.3	1.0	95.4(6.1)	95.3(4.3)	94.5(2.4)	0.9995	0.3	1.0	105.1(5.1)	87.8(6.4)	88.5(7.4)
Nuarimol	0.9937	0.3	1.0	102.4(6.2)	87.4(5.3)	101.5(5.6)	0.9980	0.3	1.0	92.2(6.6)	93.1(6.7)	103.4(6.2)
Diclofop-methyl	0.9945	0.5	2.0	94.3(9.2)	84.3(5.4)	81.5(7.7)	0.9925	0.5	2.0	87.2(7.2)	99.1(8.3)	94.3(6.2)
Piperonyl butoxide	0.9935	0.3	1.0	104.3(5.1)	89.4(4.2)	91.7(5.3)	0.9919	0.3	1.0	103.1(6.1)	101.5(4.6)	93.1(8.2)
Oxycarboxin	0.9968	1.0	3.0	104.2(6.4)	107.2(7.4)	101.4(4.3)	0.9950	3.0	10.0	102.4(4.2)	103.4(4.1)	103.3(8.1)
Resmethrin	0.9968	0.3	1.0	101.4(6.1)	109.4(9.1)	82.55(6.1)	0.9918	0.3	1.0	79.5(9.4)	80.5(7.7)	86.3(5.5)
Zoxamide	0.9992	0.3	1.0	105.3(8.1)	76.5(8.1)	81.0(7.3)	0.9942	0.3	1.0	102.1(9.3)	95.5(6.1)	93.2(9.2)
Mefenpyr-diethyl	0.9987	0.3	1.0	94.5(7.2)	110.2(8.1)	107.2(5.8)	0.9991	0.5	2.0	95.1(6.3)	98.4(7.5)	97.2(5.2)
Benzoylprop-ethyl	0.9942	1.0	3.0	107.1(7.5)	87.3(7.4)	97.2(6.2)	0.9990	1.0	3.0	91.4(4.5)	92.3(6.3)	105.1(7.1)
Spiromesifen	0.9922	1.0	3.0	101.3(5.2)	79.2(9.1)	96.5(7.8)	0.9926	1.0	3.0	106.6(8.3)	104.1(7.6)	102.1(5.2)
Endrin ketone	0.9994	0.5	2.0	96.5(5.5)	83.4(9.2)	80.2(9.1)	0.9940	0.5	2.0	94.1(8.3)	95.3(6.5)	93.4(9.3)
Fenamiphos sulfone	0.9945	1.0	3.0	104.3(7.4)	80.5(9.6)	95.4(5.2)	0.9989	1.0	3.0	108.6(5.7)	102.2(5.1)	109.2(7.3)
Bromuconazole	0.9926	0.3	1.0	107.4(7.4)	85.5(9.3)	96.4(5.5)	0.9902	0.3	1.0	79.4(11.1)	103.1(7.3)	104.1(7.2)
Fenpiclonil	0.9942	1.0	3.0	95.1(6.3)	93.2(9.1)	106.3(9.1)	0.9968	1.0	3.0	83.4(9.1)	79.4(9.3)	94.5(6.4)
Phosmet	0.9998	0.5	2.0	89.1(8.2)	74.3(9.4)	96.2(8.3)	0.9920	0.5	2.0	82.8(8.6)	1054(9.1)	102.5(9.6)
Bromopropylate	0.9964	0.5	2.0	112.4(6.5)	110.6(9.7)	113.5(6.6)	0.9971	0.5	2.0	114.1(7.2)	114.3(4.4)	113.8(6.9)
Tetramethrin	0.9903	0.5	2.0	85.0(7.1)	107.2(9.3)	117.2(7.1)	0.9932	0.5	2.0	93.7(9.2)	118.1(8.4)	123.4(9.5)
Picolinafen	0.9962	0.5	2.0	95.4(6.1)	80.2(9.4)	92.1(9.2)	0.9972	0.5	2.0	111.6(7.7)	103.8(8.9)	105.1(7.4)
Bifenthrin	0.9929	1.0	3.0	122.3(9.4)	120.5(9.6)	120.4(8.5)	0.9957	10	3.0	121.3(9.1)	121.2(9.3)	120.7(8.2)
Piperophos	0.9996	1.0	3.0	84.6(7.3)	75.8(9.2)	106.6(7.7)	0.9927	3.0	10.0	101.3(5.3)	84.4(9.5)	94.0(8.1)
4,4'-Methoxychlor	0.9914	0.3	1.0	90.5(8.4)	99.5(9.1)	96.9(7.2)	0.9957	0.3	1.0	85.5(4.6)	90.7(8.8)	105.2(4.3)
Bifenazate	0.9983	0.5	2.0	105.3(7.1)	81.2(9.5)	98.6(7.7)	0.9998	0.5	2.0	97.5(9.2)	103.4(8.5)	106.5(7.4)
Fenpropathrin	0.9902	1.0	3.0	92.5(6.6)	90.4(4.2)	94.5(5.2)	0.9924	3.0	10.0	81.6(9.5)	92.3(6.1)	94.5(5.3)
Etoxazole	0.9967	0.3	1.0	93.4(6.1)	94.2(4.5)	100.2(6.3)	0.9984	0.3	1.0	90.4(4.3)	97.2(5.3)	96.9(6.2)
Tebufenpyrad	0.9908	3.0	10.0	103.4(4.5)	75.6(9.7)	107.9(8.1)	0.9957	3.0	10.0	94.5(6.6)	83.7(8.8)	84.2(8.3)

Fenamidone	0.9971	0.3	1.0	104.2(7.3)	87.4(8.2)	104.5(7.1)	0.9930	0.3	1.0	84.5(8.4)	95.3(8.2)	101.4(5.3)
Dicofol	0.9944	0.5	2.0	109.5(7.6)	92.7(4.8)	96.9(8.0)	0.9970	0.5	2.0	87.5(4.6)	104.7(5.8)	93.3(8.2)
Metconazole	0.9952	1.0	3.0	120.8(9.5)	122.1(9.7)	118.1(7.3)	0.9909	1.0	3.0	113.5(4.2)	113.3(7.3)	120.3(9.3)
Fenazaquin	0.9996	0.3	1.0	101.1(4.3)	94.4(6.3)	103.1(4.2)	0.9916	0.5	2.0	102.3(9.6)	94.4(7.2)	95.5(4.2)
Tetradifon	0.9954	1.0	3.0	85.3(4.4)	84.5(8.1)	105.5(7.2)	0.9902	1.0	3.0	96.4(7.1)	92.5(8.3)	107.3(5.2)
Furathiocarb	0.9919	0.5	2.0	94.2(8.2)	97.1(4.4)	113.4(7.2)	0.9904	0.5	2.0	98.2(7.3)	95.8(6.6)	108.6(8.3)
Phosalone	0.9925	0.3	1.0	104.5(4.4)	83.5(5.3)	97.3(9.1)	0.9957	0.3	1.0	78.5(7.2)	104.1(4.4)	109.4(9.5)
Pyriproxyfen	0.9983	0.5	2.0	92.3(8.2)	102.4(9.4)	101.3(4.4)	0.9926	0.5	2.0	97.5(9.2)	92.5(8.4)	95.3(6.4)
Mirex	0.9904	0.3	1.0	107.5(9.4)	96.5(7.1)	88.2(4.5)	0.9903	0.3	1.0	92.4(8.1)	103.3(8.5)	88.3(4.2)
Mefenacet	0.9916	0.5	2.0	92.3(7.4)	98.4(7.2)	94.2(8.2)	0.9907	0.5	2.0	104.2(7.3)	96.3(7.1)	95.4(6.3)
Cyhalothrin	0.9934	0.3	1.0	103.5(9.5)	78.4(9.1)	80.2(7.4)	0.9926	0.3	1.0	114.1(6.4)	113.5(9.1)	92.2(7.3)
Tralkoxydim	0.9960	3.0	10.0	110.3(8.1)	97.2(9.4)	99.3(6.4)	0.9965	3.0	10.0	92.9(9.2)	113.1(8.2)	96.3(7.3)
Fenarimol	0.9904	1.0	3.0	80.2(9.2)	72.8(9.2)	92.1(9.1)	0.9932	1.0	3.0	102.3(4.2)	121.3(9.2)	99.3(8.3)
Trifenmorph	0.9981	0.5	2.0	95.2(6.3)	90.4(9.5)	88.6(8.2)	0.9982	0.5	2.0	87.8(4.4)	97.9(6.3)	77.6(10.3)
Azinphos-ethyl	0.9967	1.0	3.0	96.5(5.4)	80.3(4.3)	102.4(8.2)	0.9902	1.0	3.0	95.4(6.7)	108.5(9.6)	109.5(9.6)
Pyrazophos	0.9992	3.0	10.0	95.5(8.6)	92.3(8.5)	100.5(5.2)	0.9925	3.0	10.0	89.6(7.5)	111.4(6.3)	95.5(9.0)
Acrinathrin	0.9996	1.0	3.0	81.4(4.4)	93.5(8.6)	80.4(9.8)	0.9902	3.0	10.0	95.4(6.2)	110.3(4.6)	86.7(6.8)
Fluoroglycofen-ethyl	0.9977	3.0	10.0	95.1(6.3)	97.2(7.4)	99.3(9.1)	0.9940	3.0	10.0	92.7(7.8)	113.1(6.3)	96.5(8.6)
Fenoxaprop-ethyl	0.9909	3.0	10.0	88.8(9.6)	88.4(6.3)	84.5(6.4)	0.9928	3.0	10.0	91.6(6.7)	82.5(8.2)	82.8(7.2)
Bitertanol	0.9993	3.0	10.0	105.5(9.2)	103.4(8.9)	104.4(7.5)	0.9923	3.0	10.0	105.1(7.4)	108.5(8.2)	108.1(8.2)
Spirodiclofen	0.9923	1.0	3.0	82.8(5.1)	89.3(2.2)	92.3(6.2)	0.9962	3.0	10.0	83.3(3.1)	82.4(5.2)	89.2(5.3)
Permethrin	0.9995	1.0	3.0	88.2(8.4)	82.3(9.3)	95.3(7.1)	0.9913	1.0	3.0	103.4(7.1)	81.6(6.6)	93.2(6.1)
Pyridaben	0.9987	0.3	1.0	92.5(6.6)	93.5(5.4)	86.7(7.5)	0.9953	0.3	1.0	103.2(6.2)	84.5(6.4)	95.3(4.5)
Fluquinconazole	0.9972	0.3	1.0	105.5(7.3)	105.2(4.4)	94.5(5.3)	0.9988	0.3	1.0	79.8(8.2)	88.4(7.1)	98.5(7.2)
Coumaphos	0.9987	0.3	1.0	95.3(6.1)	95.6(4.4)	94.5(4.6)	0.9923	0.3	1.0	105.6(8.1)	88.2(6.4)	88.5(7.2)

Prochloraz	0.9997	0.3	1.0	102.3(5.4)	87.5(7.4)	101.2(5.5)	0.9978	0.3	1.0	92.5(6.2)	93.4(6.4)	103.1(8.3)
Butafenacil	0.9977	0.5	2.0	93.2(7.2)	84.1(6.2)	81.2(7.3)	0.9997	0.5	2.0	85.3(7.2)	98.3(8.4)	93.2(7.3)
Prallethrin	0.9979	0.5	2.0	106.1(9.3)	106.1(6.4)	94.3(5.1)	0.9993	0.5	2.0	95.8(6.2)	87.3(6.4)	88.1(9.2)
Cyfluthrin	0.9946	0.3	1.0	104.5(7.1)	89.4(5.2)	91.6(6.3)	0.9909	0.5	2.0	103.3(9.5)	102.1(6.3)	93.5(8.3)
Cypermethrin	0.9970	1.0	3.0	88.2(5.3)	87.5(5.2)	101.4(5.2)	0.9962	3.0	10.0	72.9(6.2)	93.2(6.1)	103.5(8.4)
Boscalid	0.9921	0.3	1.0	101.3(4.2)	108.3(9.2)	82.5(8.2)	0.9923	0.3	1.0	79.9(9.6)	80.1(9.4)	86.9(5.3)
Quizalofop-ethyl	0.9933	0.3	1.0	105.3(9.2)	76.8(9.1)	80.5(9.3)	0.9938	0.3	1.0	103.5(9.2)	95.2(6.1)	93.4(8.2)
Flucythrinate	0.9974	0.3	1.0	114.5(6.1)	112.6(8.2)	113.6(5.1)	0.9931	0.5	2.0	114.4(6.4)	110.4(8.2)	111.7(5.3)
Etofenprox	0.9933	1.0	3.0	108.5(7.1)	87.4(6.3)	97.5(8.1)	0.9992	1.0	3.0	91.5(4.9)	92.6(6.2)	105.3(8.1)
Pyridalyl	0.9936	1.0	3.0	101.2(6.2)	79.1(9.1)	96.7(7.2)	0.9999	1.0	3.0	107.4(9.6)	105.5(7.1)	102.2(7.2)
Fenvalerate	0.9981	0.5	2.0	122.2(3.2)	120.3(9.1)	121.1(9.1)	0.9958	0.5	2.0	120.1(8.4)	120.4(6.2)	120.1(7.2)
Flumioxazin	0.9956	1.0	3.0	104.1(7.3)	80.2(7.7)	95.8(6.2)	0.9999	1.0	3.0	108.5(6.3)	102.5(9.1)	109.3(7.2)
Pyraclostrobin	0.9935	3.0	10.0	115.4(7.1)	118.0(6.2)	112.3(5.8)	0.9908	3.0	10.0	113.5(6.2)	111.4(9.2)	112.1(7.1)
tau-Fluvalinate	0.9910	3.0	10.0	103.8(5.1)	104.2(9.3)	104.1(6.3)	0.9993	3.0	10.0	105.8(6.2)	109.4(10.2)	109.1(9.5)
Difenoconazole	0.9929	1.0	3.0	114.2(3.2)	118.3(8.2)	114.3(5.2)	0.9913	3.0	10.0	114.3(9.1)	116.4(7.2)	119.7(4.3)
Deltamethrin	0.9916	1.0	3.0	98.2(6.2)	83.1(8.3)	96.2(7.0)	0.9970	1.0	3.0	102.2(8.1)	81.8(6.8)	88.4(6.1)
Azoxystrobin	0.9974	0.3	1.0	116.2(5.9)	111.1(4.2)	120.3(7.1)	0.9973	0.3	1.0	123.4(6.2)	124.2(5.2)	108.5(4.0)
Dimethomorph	0.9923	0.3	1.0	122.4(6.1)	120.1(4.0)	120.9(5.3)	0.9925	0.3	1.0	120.8(9.2)	121.2(5.1)	121.2(6.3)
